# Supplementary material for: Extracellular vesicle engineering using a small scaffold protein
Source: Nat Commun. 2026 Mar 10;17:3726. doi: 10.1038/s41467-026-70451-x (PMC13102933; doi:10.1038/s41467-026-70451-x)
Supplement: Supplementary file 1 — Supplementary Information [file 41467_2026_70451_MOESM1_ESM.pdf]

Supplementary Information

Extracellular vesicle engineering using a small scaffold protein

Wenjing Yan[1,2,#], Shizhi Wang[1,#,*], Haibin Hao[3,#], Hong Lin[4], Chen Wang[5], Shuqian Xie[1], Xing Zhang[1], Yiran Lu[1], Xin Ding[1], Xue Chen[1], Haohan Liu[1], Guiyuan Zhang[6], Dong Wei[6], ChangYan Ma[7], Cheng Tang[8], Xiuting Li[9], Bingjia Yu[9], Jing Hu[1], Zhongze Gu[6], Evan Yi-Wen Yu[10,*], Weiqin Li[3,*], Jiang Xia[11,*], Hao Zhang[3,6,12,*]

[1] Key Laboratory of Environmental Medicine Engineering, Ministry of Education, School of Public Health, Southeast University, Nanjing, China.

[2] School of Public Health, Shandong Second Medical University, Weifang 261053, China.

[3] Department of Critical Care Medicine, Jinling Hospital, Affiliated Hospital of Medical School, Nanjing University

[4] School of Medicine, Southeast University, Nanjing 210009, China

[5] School of Pharmacy, Jiangsu University, Zhenjiang, Jiangsu 212013, China.

[6] State Key Laboratory of Digital Medical Engineering, School of Biological Science and Medical Engineering, Southeast University, Nanjing, China.

[7] Department of Medical Genetics, Nanjing Medical University, 101 Longmian Avenue, Nanjing, China.

[8] Department of Orthopaedics, Nanjing First Hospital, Nanjing Medical University, Nanjing, China.

[9] School of Public Health Administration, Jiangsu Health Vocational College, No. 69, Huangshanling Road, Pukou District, Nanjing, China.

[10] Key Laboratory of Environmental Medicine and Engineering of Ministry of Education, and Department of Epidemiology & Biostatistics, School of Public Health, Southeast University, Nanjing, China.

[11] Department of Chemistry, the Chinese University of Hong Kong, Shatin, Hong Kong SAR, China.

[12] EVLiXiR Biotech Inc., Nanjing 210032, Jiangsu, China.

[#] Contributed equally.

* Corresponding authors: shizhiwang2009@seu.edu.cn (Shizhi Wang), evan.yu@maastrichtuniversity.nl (Evan Yi-Wen Yu), liweiqindr@nju.edu.cn (Weiqin Li), jiangxia@cuhk.edu.hk (Jiang Xia), and scottzhang09@gmail.com (Hao Zhang).

Contents

Items	Page No.
**Figure S1.** Characterization of Expi293F-EVs	3
**Figure S2.** Generation of stable scaffold protein-expressing cell lines and size analysis of engineered EVs.	4
**Figure S3.** Expression of EGFP proteins in engineered EVs	5
**Figure S4.** Quantitative analysis of the expression of EGFP protein in engineered EVs	6
**Figure S5.** Cellular uptake characteristics of ENPP1.	7
**Figure S6.** Transfection efficiency of ENPP1 truncated variant in Expi293F cells and expression of FLAG protein in EVs.	8
**Figure S7.** EN144 loading efficiency in various cells and their EVs.	9
**Figure S8.** Phenotypic responses of diverse cell types to varying doses of EN144-EVs.	10
**Figure S9.** Safety of EN144-EVs based on hematological and biochemical indices through intravenous administration to mice.	11
**Figure S10.** Effects of EN144-EVs administration on mouse organ pathology.	13
**Figure S11.** Design and characterization of chondrocyte-targeted EVs.	14
**Figure S12.** Blocking effect of CAP peptide on the interaction between ENPP1-EV^{CAP} and chondrocytes.	15
**Figure S13.** Loading and delivery of EGFP mRNA within EVs.	16
**Figure S14.** Loading of CRISPR/Cas9 Complex into EVs.	18
**Figure S15.** Establishment and verification of *in vitro* and *in vivo* inflammation models.	19
**Figure S16.** Loading capacity assessment and anti-inflammatory evaluation of EN144-EV^{hgp130}.	20
**Figure S17.** EN144-EV^{mgp130} targets the IL-6 trans-signaling pathway.	21
**Figure S18.** Construction and targeting assessment of EN144-EV$^{hgp130\text{-}CAP}$ and EN144-EV^{hgp130}.	22
**Figure S19.** Effect of EN144-EV$^{hgp130\text{-}CAP}$ on osteoarthritis (OA)-like chondrocyte phenotype.	23
**Figure S20.** Modulation of synovial gene expression by EN144-EV$^{hgp130\text{-}CAP}$.	24
**Figure S21.** Assessment of toxic effects on vital organs across treatment groups.	26
**Supplementary Table 1.** Information on the use of antibodies.	28
**Supplementary Table 2.** Amino acid sequence of the various genetic constructs used in this study.	29
**Supplementary Table 3.** Sequence information of primers.	34
**Supplementary Table 4.** Expression of candidate scaffold proteins in Expi293F-EVs.	35
**Supplementary Table 5.** Efficiency of Indel Generation calculated with TIDE.	36

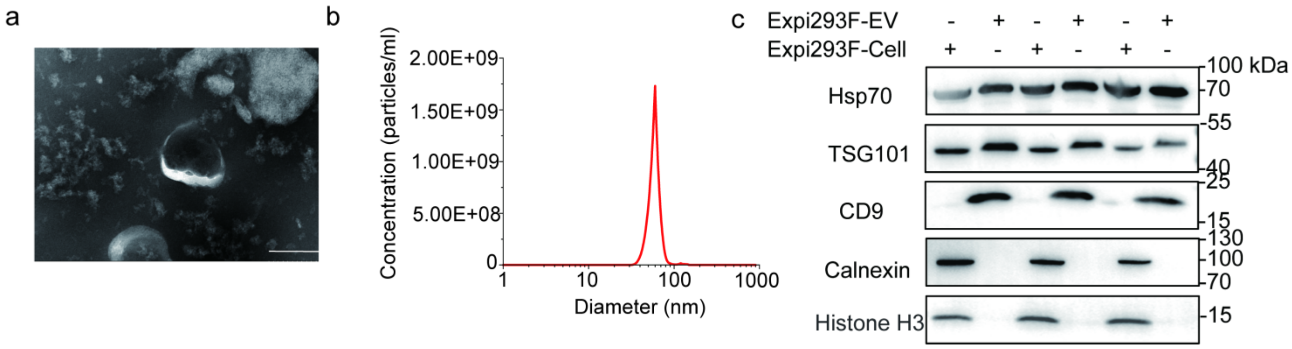

Figure S1. Characterization of Expi293F-EVs. (a) Representative transmission electron microscopy (TEM) image of Expi293F-derived EVs (Expi293F-EVs), scale bar = 100 nm. (b) Particle size distribution of Expi293F-EVs based on Resistive Pulse Sensing (RPS) analysis. (c) Expression of Hsp70, TSG101, CD9, calnexin and histone H3 in Expi293F cell lysates and Expi293F-EVs. Each sample contains 10 μg of cell lysate or 3.0×10^9 EV particles. Source data are provided as a Source Data file.

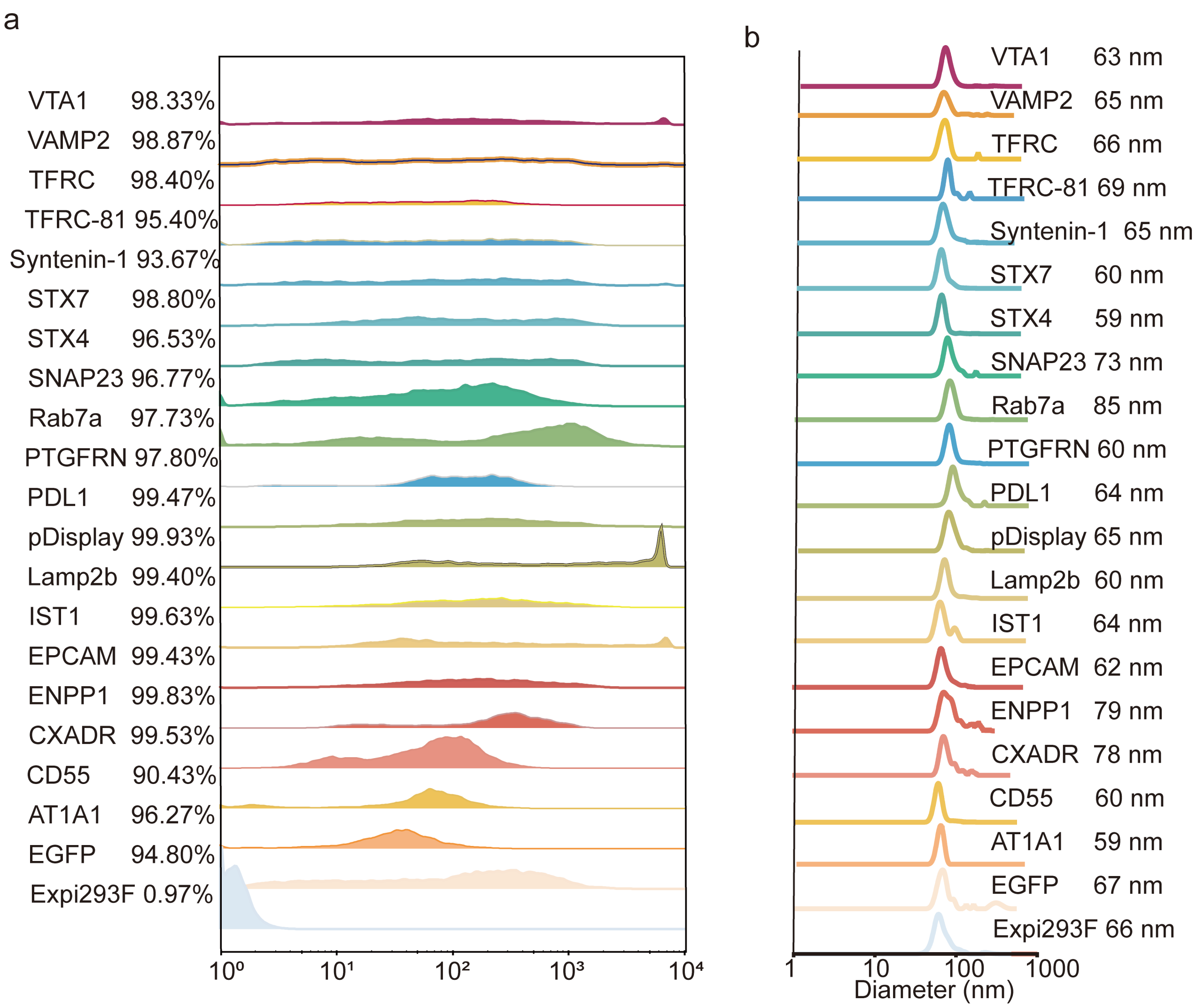

Figure S2. Generation of stable scaffold protein-expressing cell lines and size analysis of engineered EVs. (a) Flow cytometric analysis showing the percentages of EGFP-positive cells among stable scaffold protein-expressing cell lines. TFRC-81 represents the transmembrane sequence of TFRC; Syntenin-1 represents the transmembrane sequence of Syntenin-1. (b) Particle size distribution of various engineered EVs based on RPS analysis.

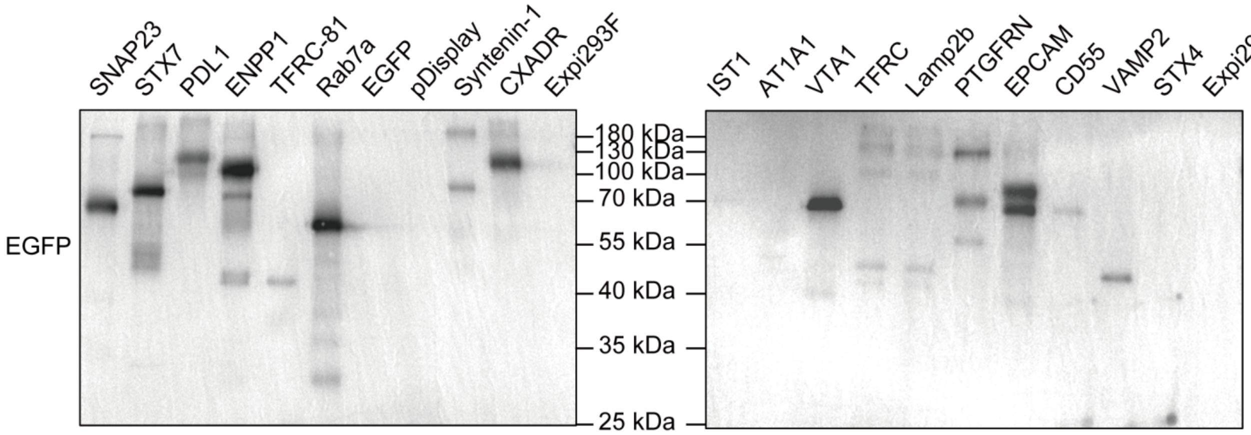

Figure S3. Expression of EGFP proteins in engineered EVs. A panel of proteins was selected as candidates for new EV-sorting proteins. Class I sorting proteins include STX4, ENPP1, TFRC, TFRC-81 (transmembrane sequence of TFRC), VAMP2, and CD55, inserted into the pEGFP-C1 vector. Class II sorting proteins include EPCAM, AT1A1, Syntenin-1, CXADR, PDL1, STX7, IST1, SNAP23, VTA1, PTGFRN, and Lamp2b, inserted into the pCDNA3.1-EGFP vector. EV-sorting protein Rab7a is also included. Briefly, EVs were collected from transfected cells, and EGFP levels of the overexpressed sorting proteins in the purified EVs were analyzed by western blot analysis (3.0 × 10^9 particles). Source data are provided as a Source Data file.

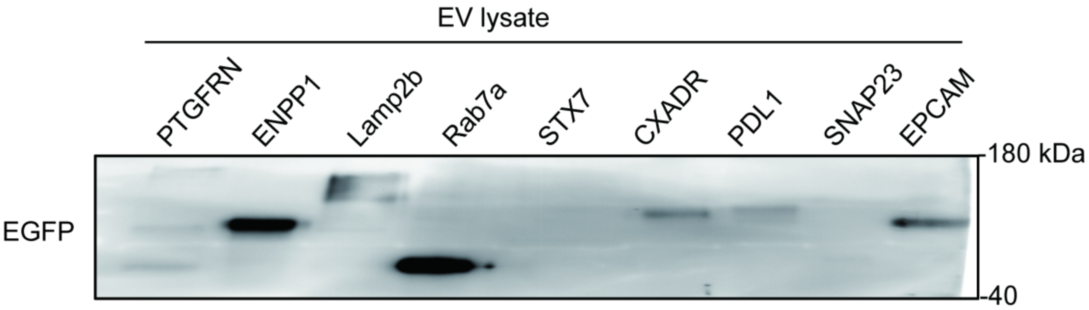

Figure S4. Semi-quantitative analysis of EGFP protein in engineered EVs. EGFP protein levels in engineered EVs were detected by Western blot using an anti-GFP antibody. A consistent amount of EVs (3.0×10^9 particles per lane) was loaded for each sample. Grayscale intensity analysis of the target band was performed using Image J software. Source data are provided as a Source Data file.

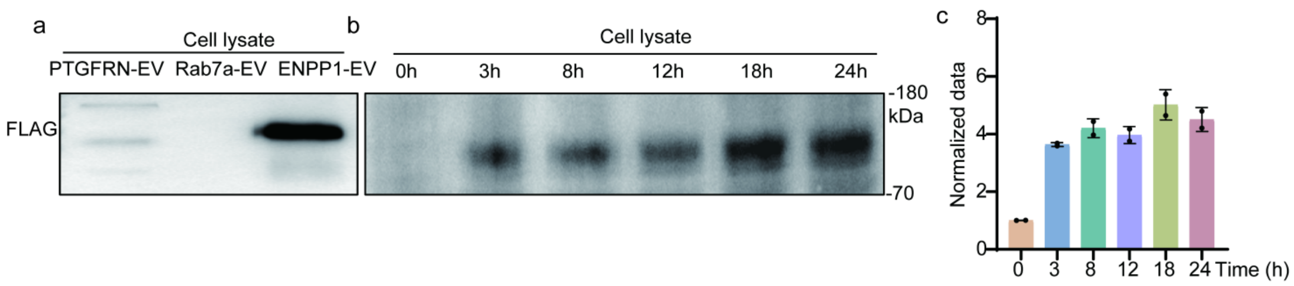

Figure S5. Cellular uptake characteristics of ENPP1. (a) Expression of FLAG-tagged proteins in Expi293F cells co-cultured with Rab7a-EVs, ENPP1-EVs, or PTGFRN-EVs by western blot analysis. Each sample contains 1.0×10^{10} particles EVs. (b-c) Time-course analysis of FLAG-tagged proteins in Expi293F cells exposed to ENPP1-EVs (b) and corresponding quantitative assessment (c). Each sample contains 1.0×10^{10} particles EVs. The data are presented as mean ± SD. Source data are provided as a Source Data file.

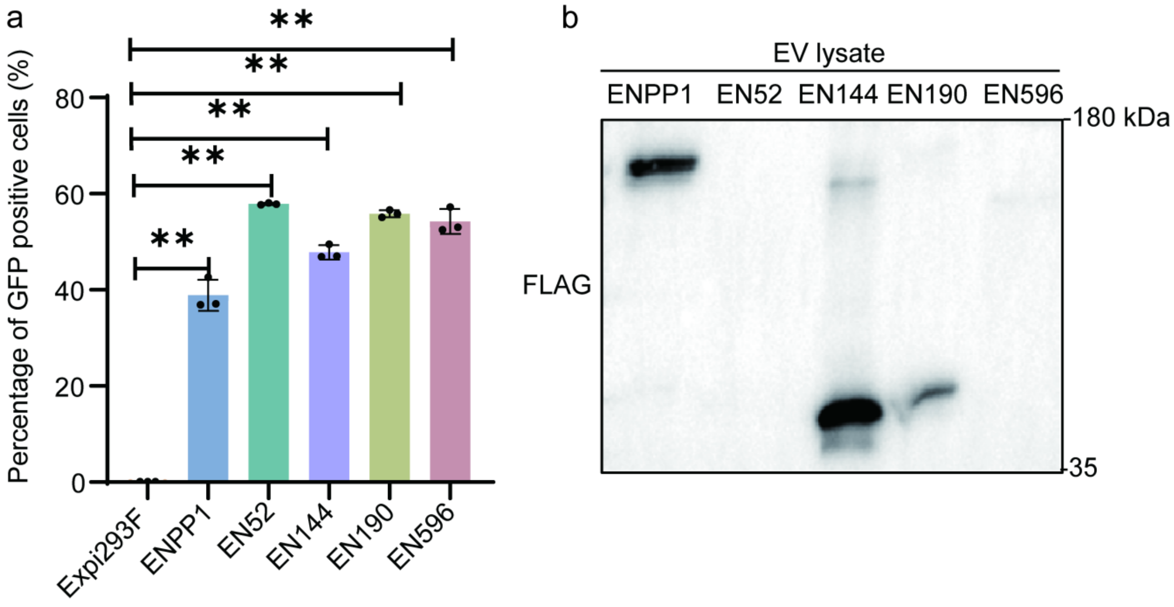

Figure S6. Transfection efficiency of ENPP1 truncated variant in Expi293F cells and expression of FLAG protein in EVs. (a) Flow cytometric analysis showing the percentages of EGFP-positive cells among Expi293F cells transfected with plasmids encoding different ENPP1 truncated variants. n = 3 in each group. ****$P < 0.0001$. (b) FLAG-tagged protein in engineered EVs was semi-quantitatively analyzed by Western blot using an anti-FLAG antibody, with a standardized load of 3.0×10^9 EV particles per lane. Grayscale intensity analysis of the target band was performed using Image J software. n = 3 in each group. The data are presented as mean ± SD. The p values (a) were analyzed by one-way ANOVA followed by Dunnett's multiple comparisons test. **$P < 0.01$. Source data and exact p-value are provided as a Source Data file.Source data are provided as a Source Data file.

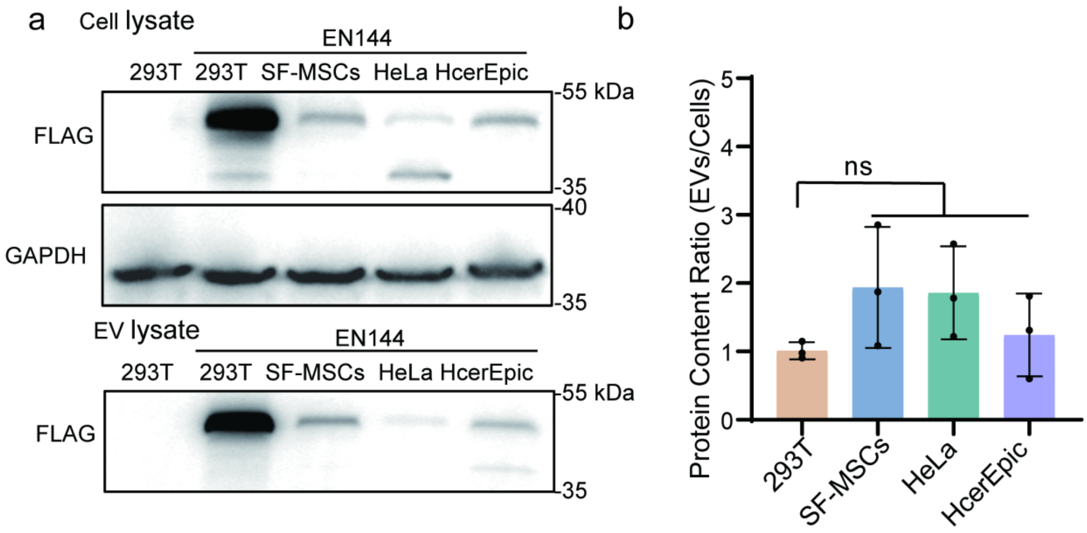

Figure S7. EN144 loading efficiency in various cells and their EVs. (a) Expression of FLAG-tagged proteins in cell lysates (top) and EVs (bottom) from 293T, SF-MSCs, HeLa, or HcerEpic cells transfected with EN144 plasmid. Top panel: Equal amounts of cell lysates were loaded as follows: lane 1, cell lysates from untransfected 293T cells (control); lanes 2-5, cell lysates from 293T, SF-MSCs, HeLa, or HcerEpic cells transfected with EN144 plasmid. Bottom panel: Equal amounts of EVs (3.0×10^9 particles EVs) were loaded as follows: lane 1, EV lysates from untransfected 293T cells (control); lanes 2-5, EV lysates from 293T, SF-MSCs, HeLa, or HcerEpic cells transfected with EN144 plasmid. n = 3 in each group. (b) FLAG-tagged protein content ratio of EVs-to-Cells in EN144-transfected 293T, SF-MSCs, HeLa, or HCerEpiC cells based on the gray value of western blot band in (a). Consistent FLAG protein expression across all tested cell lines and their EVs confirmed the efficient loading capability of EN144 in diverse cellular contexts. One-way ANOVA accompanied by Tukey's post-hoc test was employed. Data are analyzed of three independent experiments and shown as mean ± SD. ns $P > 0.05$. The data are presented as mean ± SD. The p values (b) were analyzed by one-way ANOVA followed by Dunnett's multiple comparisons test. ns $P > 0.05$. Source data and exact p-value are provided as a Source Data file.

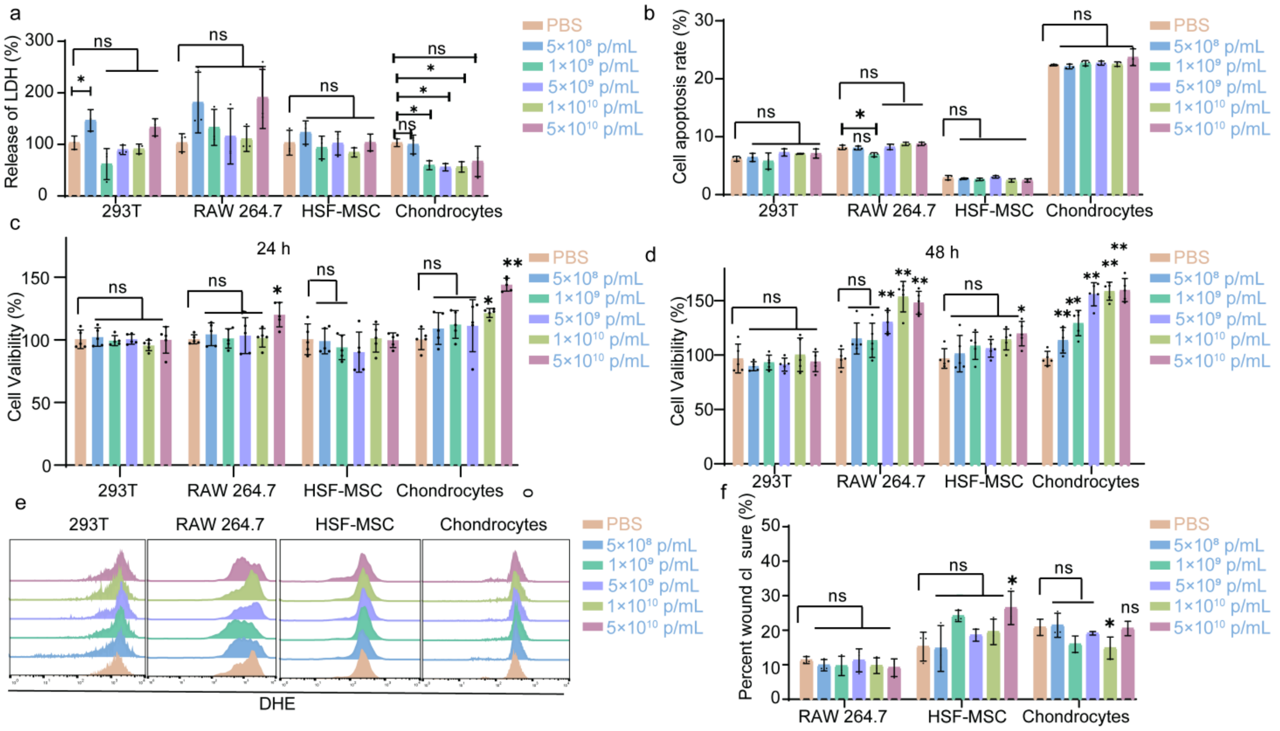

Figure S8. Phenotypic responses of diverse cell types to varying doses of EN144-EVs. (a-f) Analysis of doses-response of EN144-EV doses (5.0 × 10^8, 1.0 × 10^9, 5.0 × 10^9, 1.0 × 10^{10}, and 5.0 × 10^{10} particles/mL) on LDH release (a), apoptosis (b), viability at 24 h (c), viability at 48 h (d), ROS levels (e), and migration ability (f) in rat primary chondrocytes, RAW 264.7 cells, 293T cells, or SF-MSC cells. n = 3 in each group. The data are presented as mean ± SD. The p values (a, b, c, d, f) were analyzed by either one-way ANOVA or Brown-Forsythe ANOVA test, followed by Dunnett's or Dunnett's T3 multiple comparisons test. ns $P > 0.05$, *$P < 0.05$, **$P < 0.01$. Source data and exact p-value are provided as a Source Data file.

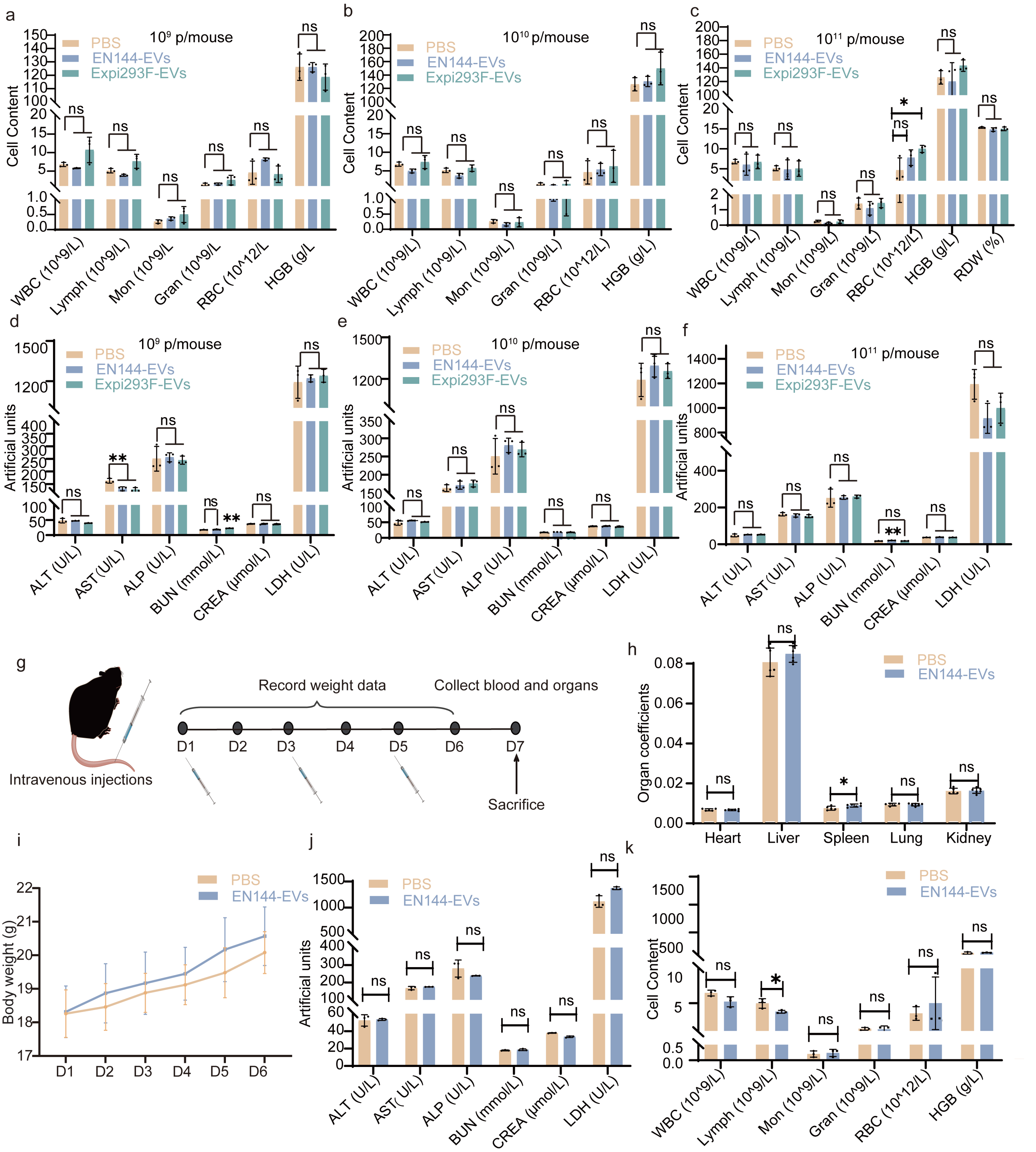

Figure S9. Safety of EN144-EVs based on hematological and biochemical indices through intravenous administration to mice. (a-c) EV injections did not cause toxicity to mice based on peripheral blood parameters (white blood cells, red blood cells, hemoglobin, granulocytes, lymphocytes, and monocytes). Blood samples were analyzed 24 hours after intravenous injection of DiR-labeled Expi293F-EVs or EN144-EVs at doses of 1.0 × 10^9, 1.0 × 10^{10}, and 1.0 × 10^{11} particles/mouse via the tail vein (n = 3 mice/group). (d-f) Safety assessment based on hepatic function markers (ALT, ALP, AST), renal function markers (BUN, CREA), and lactate dehydrogenase (LDH) 24 hours post-injection (n = 3 mice/group). Data in a-f were analyzed by one-way ANOVA followed by Tukey's post hoc test and are presented as mean ± SD from three independent experiments. (g) Schematic of the experimental design: repeated intravenous injections of 1.0 × 10^{11} particles EN144-EVs (100 μL) or an equivalent volume of PBS via the tail vein every other day for a total of three administrations. This figure was created using MedPeer

(medpeer.cn). (h) Body weight monitoring during repeated administrations (n = 3 mice/group). (i) Organ coefficients - defined as the ratio of organ weight (heart, liver, spleen, lung, and kidney) to body weight - after repeated EV administrations (n = 5 mice/group). (j-k) Hematological parameters (n = 3) and serum biochemical profiles in the repeated administration group (n = 3). The data are presented as mean ± SD. The p values (a, b, c, d, e, f) were analyzed by either one-way ANOVA or Brown-Forsythe ANOVA test, followed by Dunnett's or Dunnett's T3 multiple comparisons test. ns $P > 0.05$, *$P < 0.05$, **$P < 0.01$. Data in h-k were compared using two-tailed t-test and expressed as mean ± SD from three independent experiments. ns $P > 0.05$, *$P < 0.05$. Source data and exact p-value are provided as a Source Data file.

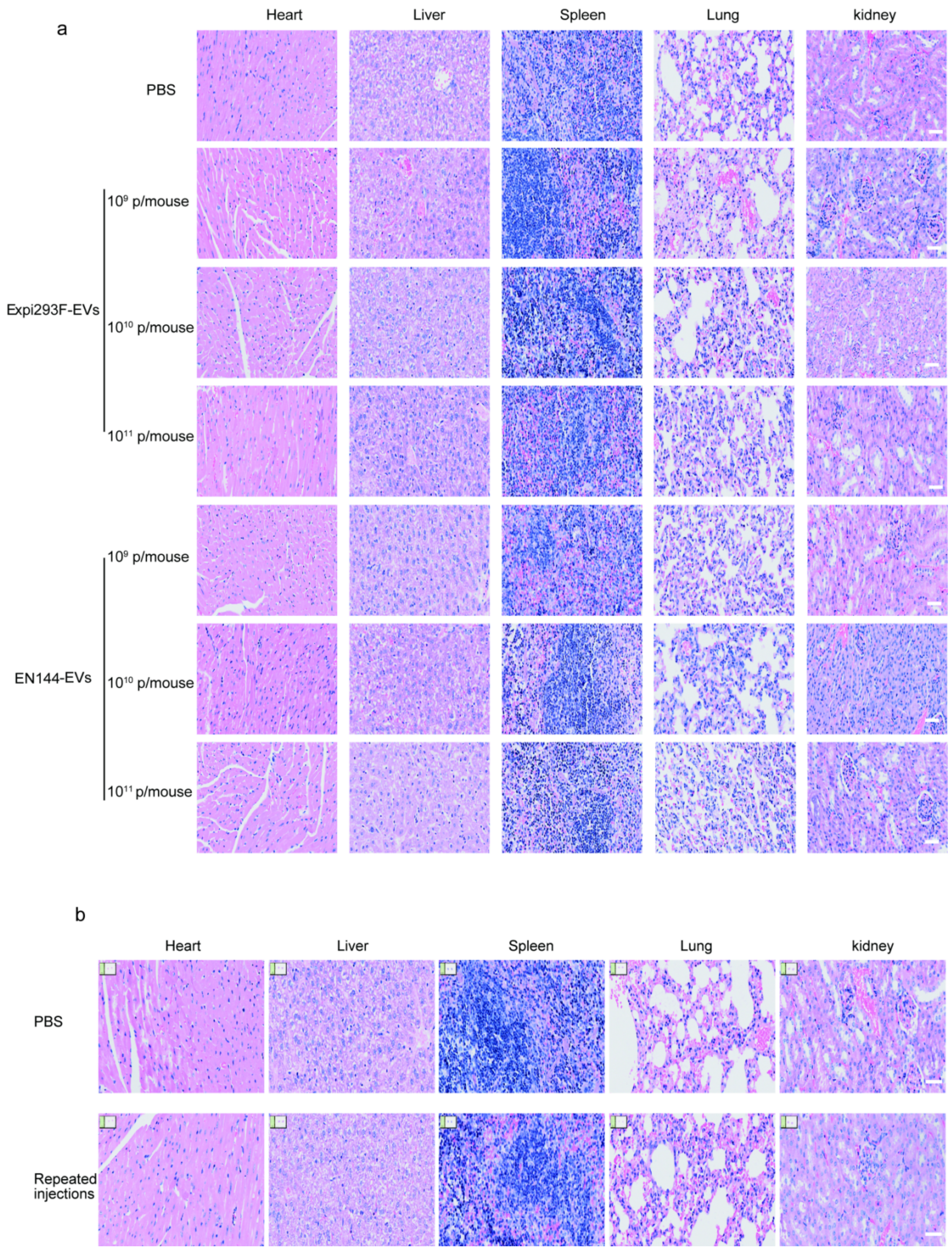

Figure S10. Effects of EN144-EVs administration on mouse organ pathology. (a) Representative images of H&E staining for histological analysis in heart, liver, spleen, lung, and kidney tissues of mice 24 hours after intravenous injection of DiR-labeled Expi293F-EVs or EN144-EVs at different doses (1.0×10^9, 1.0×10^{10}, and 1.0×10^{11} particles/mouse). Scale bars: 100 μm. (b) Representative images of H&E staining for histological analysis in heart, liver, spleen, lung, and kidney tissues of mice with repeated intravenous injections of 1.0×10^{11} particles EN144-EVs (100 μL) or an equivalent volume of PBS via the tail vein every other day for a total of three administrations. Scale bars: 100 μm.

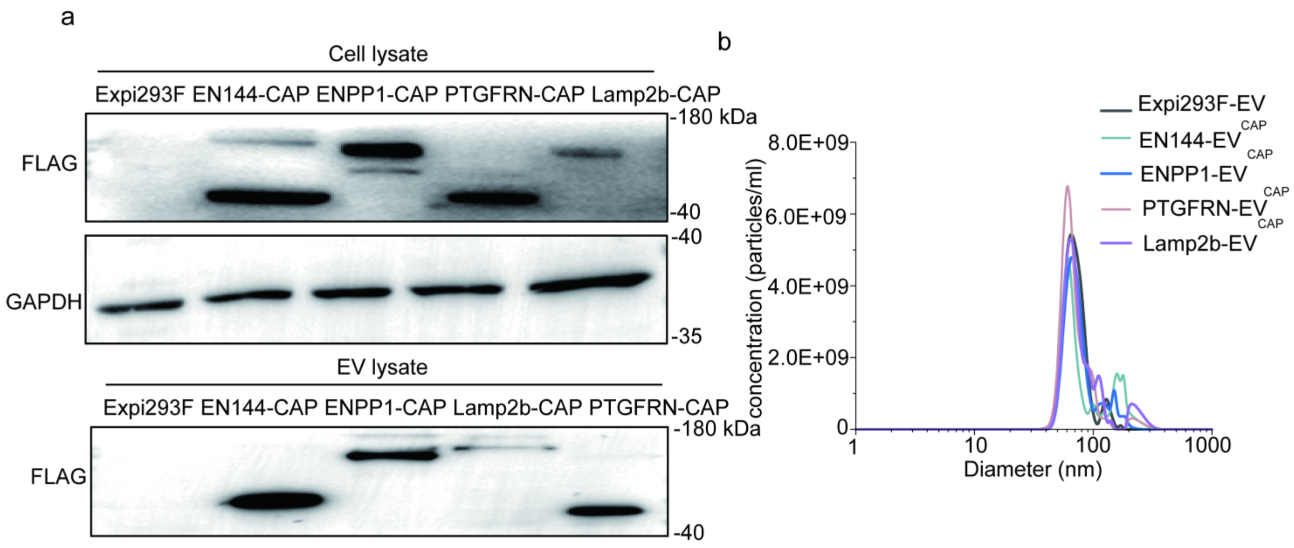

Figure S11. Design and characterization of chondrocyte-targeted EVs. (a) Expression of FLAG-tagged proteins in scaffold protein-transfected Expi293F cells and their secreted EVs by western blot analysis. Top panel: FLAG-tagged protein expression levels in lysates of Expi293F cells transfected with plasmids encoding EN144-CAP, ENPP1-CAP, PTGFRN-CAP, or Lamp2b-CAP scaffold proteins. Bottom panel: Detection of FLAG protein in EVs derived from corresponding transfected cells, demonstrating scaffold protein-dependent cargo loading into EVs. (b) Size distribution of chondrocyte-targeting EVs by RPS. All EVs exhibited a predominant diameter range of 50-150 nm, consistent with canonical EV size characteristics. Source data are provided as a Source Data file.

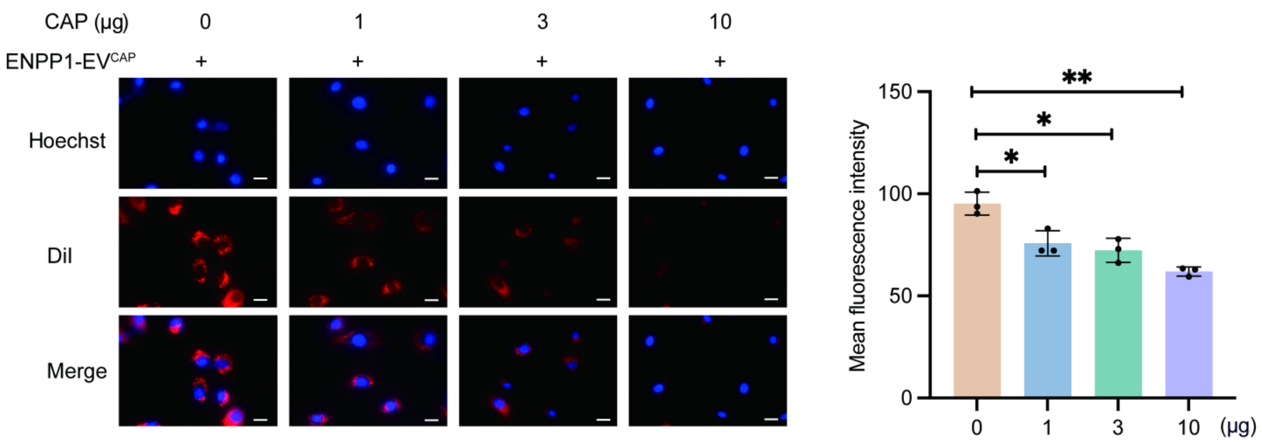

Figure S12. Blocking effect of CAP peptide on the interaction between ENPP1-EV^{CAP} and chondrocytes. Primary chondrocytes were pre-incubated with various concentrations of CAP peptide (1 μg, 3 μg, and 10 μg) for 1 hour to block cell surface receptors, following co-incubation with DiI-labeled ENPP1-EV^{CAP} for 2 hours. Left panel: Fluorescence images illustrate the internalization of ENPP1-EV^{CAP} by chondrocytes across treatment groups, demonstrating the concentration-dependent inhibitory effect of CAP peptide. Scale bar = 100 μm. Right panel: Quantitative analysis of mean fluorescence intensity in left panel (n = 3 each group). The p values were analyzed by Brown-Forsythe ANOVA test, followed by Dunnett's T3 multiple comparisons test. *P < 0.05, **P < 0.01. Source data and exact p-value are provided as a Source Data file.

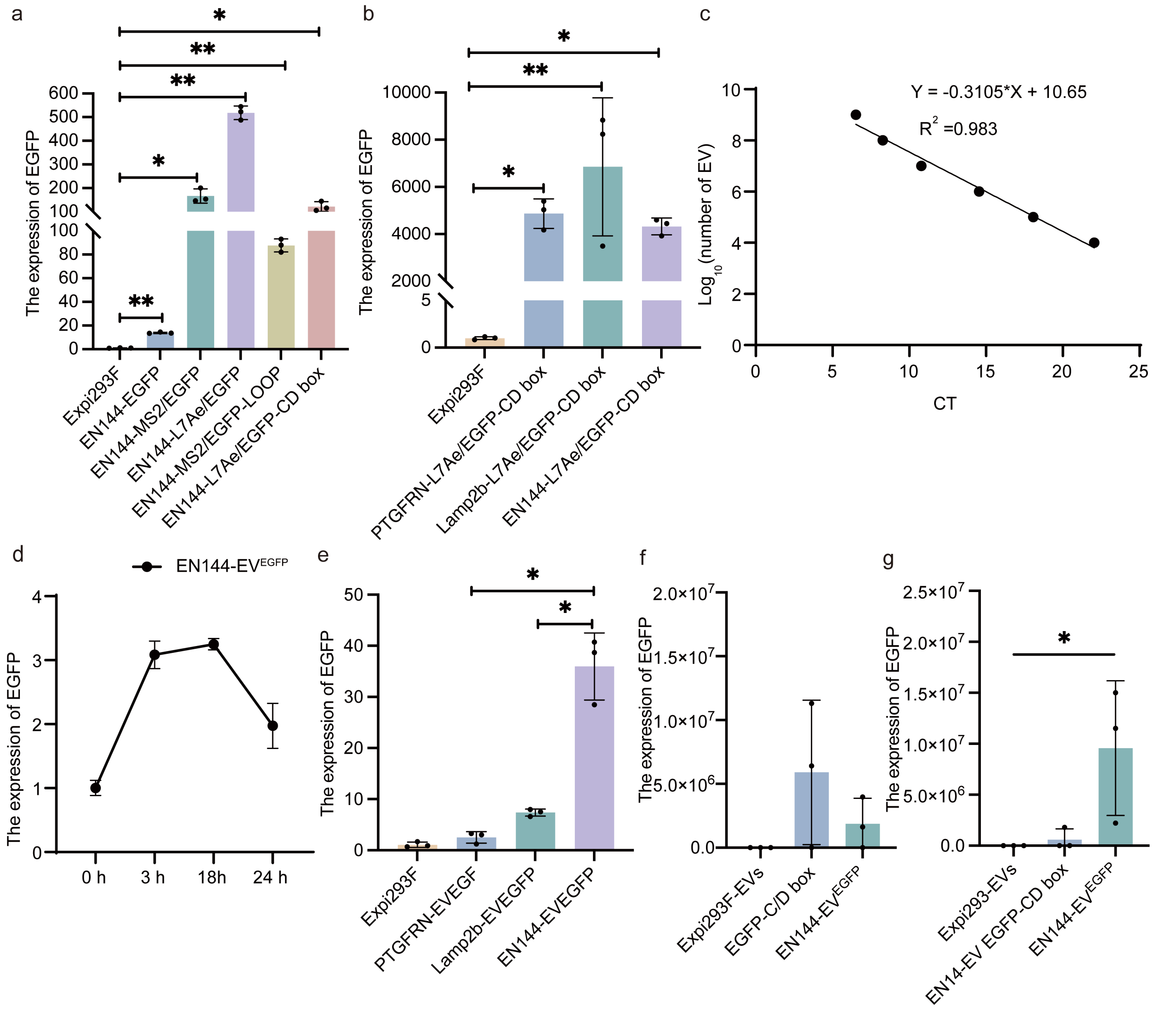

Figure S13. Loading and delivery of EGFP mRNA within EVs. (a) Four transfection schemes in Expi293F: EN144-EGFP (fusion of EN144 and EGFP), EN144-MS2/EGFP (co-transfection with EN144-MS2 plasmid and EGFP plasmid), EN144-L7Ae/EGFP (co-transfection with EN144-L7Ae plasmid and EGFP plasmid), EN144-MS2/EGFP-LOOP (co-transfection with EN144-MS2 plasmid and EGFP-LOOP plasmid), and EN144-L7Ae/EGFP-C/D box (co-transfection with EN144-L7Ae plasmid and EGFP-CD box plasmid). Relative EGFP mRNA expression levels in Expi293F cells post-transfection with EN144-EGFP, EN144-MS2/EGFP, EN144-L7Ae/EGFP, EN144-MS2/EGFP-LOOP, or EN144-L7Ae/EGFP-C/D box. n = 3 each group. (b) Relative EGFP mRNA expression levels in Expi293F cells post-transfection with L7Ae-C/D box system scaffolds (EN144, PTGFRN, or Lamp2b). n = 3 each group. (c) Standard curve construction for EGFP mRNA expression levels against EV copy numbers. (d) Comparison of EGFP mRNA expression in 293T cells co-cultured with EN144-EV^{EGFP} (1.0 × 10^{10} particles) for various time (3, 18, or 24 hours) by qRT-PCR (n = 3 each group). (e) Comparison of EGFP mRNA expression in 293T cells co-cultured with EN144-EV^{EGFP}, PTGFRN-EV^{EGFP}, or Lamp2b-EV^{EGFP} (1.0 × 10^{10} particles/group) for 18 hours by qRT-PCR. (n = 3 each group). (f) Quantification of EGFP protein in EVs using LC-MS/MS. Three types of EVs were analyzed: Expi293F-EVs, EGFP-C/D box, and EN144-EV^{EGFP}, with each sample containing 2 × 10^{10} particles. The sources of the EVs are as follows: EN144-EV^{EGFP} was derived from the supernatant of cells co-transfected with EN144-L7Ae and EGFP-C/Dbox plasmids; EGFP-CD box was obtained from the supernatant of cells transfected with the EGFP-C/D box plasmid only; and Expi293F-EVs were isolated from the supernatant of untransfected cells. (g) Cells were incubated with EN144-EV^{EGFP} (1.0 × 10^{10} particles), and intracellular EGFP protein levels were measured using LC-MS/MS (n = 3 each group). The data are presented as

mean ± SD. The p values (a, b, e, f, g) were analyzed by unpaired t test or one-way ANOVA or Brown-Forsythe ANOVA test, followed by Dunnett's or Dunnett's T3 multiple comparisons or unpaired t test with Welch's correction test. ns $P > 0.05$, *$P < 0.05$, **$P < 0.01$. Source data and exact p-value are provided as a Source Data file.

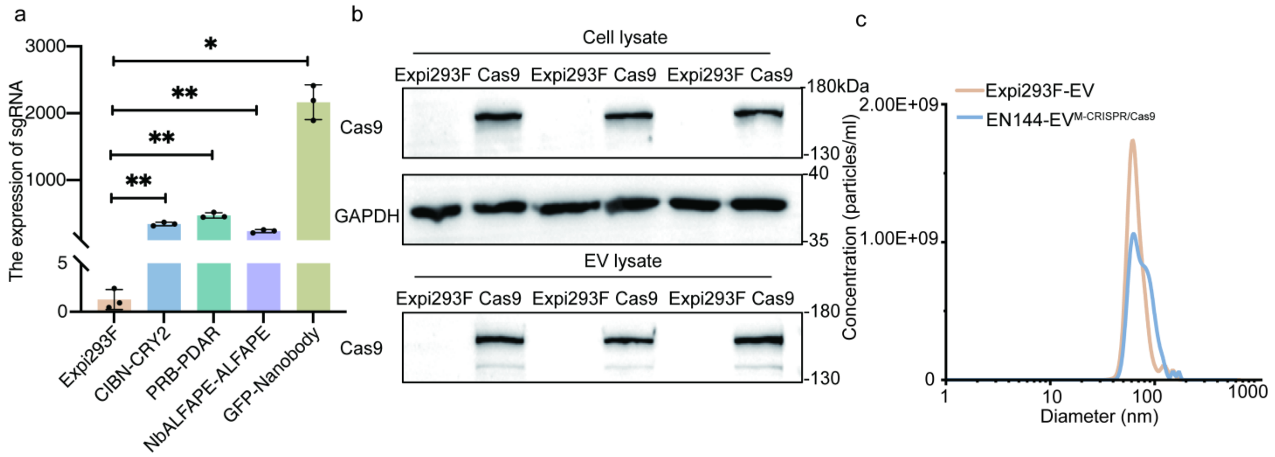

Figure S14. Loading of CRISPR/Cas9 Complex into EVs. (a) sgRNA expression levels in Expi293F cells utilizing interacting pairs (GFP-Nanobody, CIBN-CRY2, PRB-PDAR, NbALFA^{PE}-ALFA) measured by qRT-PCR. (n = 3 each group). (b) Expression levels of Cas9 protein in Expi293F cells transfected with EN144 plasmid carrying GFP-nanobody (Top panel) and EVs (EN144-EV$^{M\text{-}CRISPR/Cas9}$) isolated from transfected cells (Bottom panel). (n = 3 each group). (c) Particle size distribution of Expi293F-EVs and EN144-EV$^{M\text{-}CRISPR/Cas9}$ based on RPS analysis. The data are presented as mean ± SD. The p values (a) were analyzed by Brown-Forsythe ANOVA test, followed by Dunnett's T3 multiple comparisons test. ns $P > 0.05$, *$P < 0.05$, **$P < 0.01$. Source data and exact p-value are provided as a Source Data file.

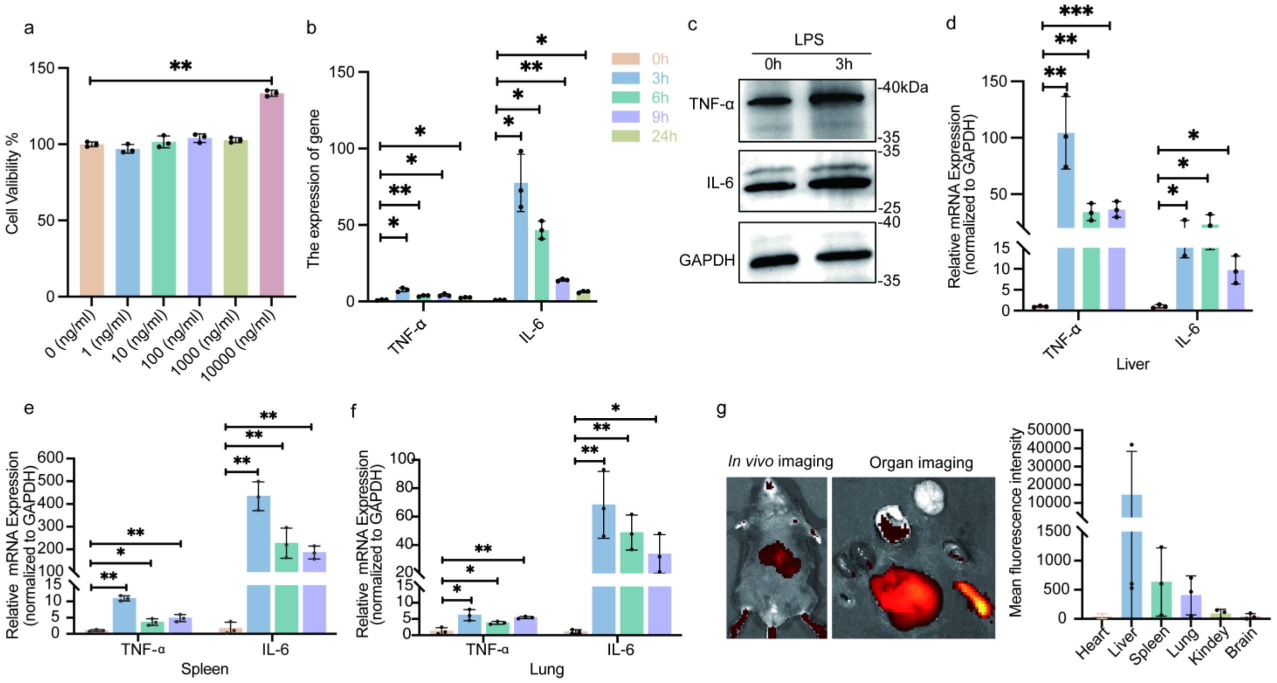

Figure S15. Establishment and verification of *in vitro* and *in vivo* inflammation models. (a) Cell viability of RAW 264.7 treated with LPS (0, 1, 10, 100, or 1000 ng/mL) for 24 hours was assessed using CCK-8 assay (n = 3 each group). (b) The mRNA levels of TNF-α and IL-6 in RAW 264.7 treated with 1000 ng/mL LPS for 0, 3, 6, 9, or 24 hours were measured by qRT-PCR. (c) Expression of TNF-α and IL-6 protein in RAW 264.7 exposed to 1000 ng/mL LPS for 0 or 3 hours were measured by WB analysis. (D-F) The mRNA levels of TNF-α and IL-6 in liver (d), spleen (e), and lung (f) tissues isolated from mice intraperitoneally injected with 10 mg/kg LPS post-injection after 3, 6, or 9 hours were quantified. n = 3 each group. (g) Biodistribution of EN144-EV^{mgp130} in septic mice. Left panel: Representative *in vivo* bioluminescence imaging and *ex vivo* tissue imaging (heart, liver, spleen, lung, kidney, and brain) 6 hours after intravenous administration of EN144-EV^{mgp130} (1.0×10^{10} particles). Right panel: Quantification of EVs in tissues based on the fluorescence signal of DiR. n = 3 each group. The data are presented as mean ± SD. The p values (a, b, d, e, f) were analyzed by either one-way ANOVA or Brown-Forsythe ANOVA test, followed by Dunnett's or Dunnett's T3 multiple comparisons test. ns $P > 0.05$, *$P < 0.05$, **$P < 0.01$. Source data and exact p-value are provided as a Source Data file.

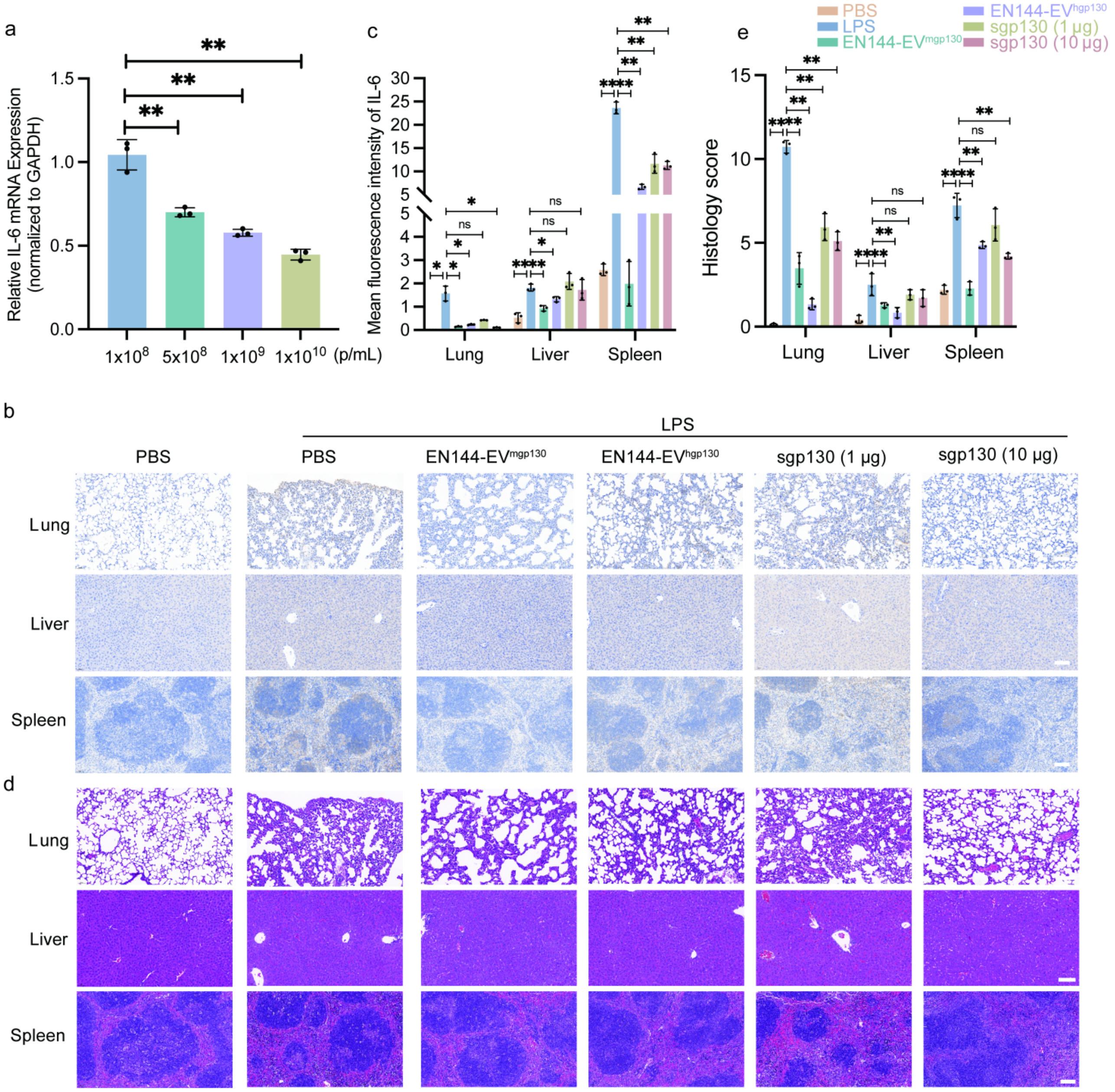

Figure S16. Loading capacity assessment and anti-inflammatory evaluation of EN144-EV^{hgp130}. (a) The mRNA levels of IL-6 in RAW 264.7 treated with 1000 ng/mL LPS following co-culture with various concentrations (1.0×10^8, 5.0×10^8, 1.0×10^9, or 1.0×10^{10} particles/mL) of EN144-EV^{hgp130} were measured by qRT-PCR. (b-e) EV preparations (EN144-EV^{hgp130} and EN144-EV$^{hgp130\text{-}CAP}$) of 1.0×10^{10} EV particles or sgp130 (1 μg or 10 μg) were injected into septic mice. The mice were sacrificed post-injection after 6 hours, and the organs were dissected for analysis. (b) Immunohistochemical (IHC) staining of IL-6 protein in lung, liver, and spleen tissues (brown: positive signals; blue: nuclear counterstaining with hematoxylin). Scale bar = 100 μm. (d) Therapeutic evaluation of intervention groups on inflammatory injury in organs of septic mice. Representative H&E stained images showing histopathological alterations in lung, liver, and spleen tissues of intervention and control groups (scale bar = 100 μm). (c) Semi-quantitative analysis of IHC-positive areas using ImageJ software based on IHC results in (b). (e) Semi-quantitative inflammatory scores (0-3: none/mild/moderate/severe injury) based on H&E staining results in (d). The data are presented as mean ± SD. The p-values (a, b, e, f, g) were analyzed by either one-way ANOVA or Brown-Forsythe ANOVA test, followed by Dunnett's or Dunnett's T3 multiple comparisons test. ns $P > 0.05$, *$P < 0.05$, **$P < 0.01$. Source data and exact p-value are provided as a Source Data file.

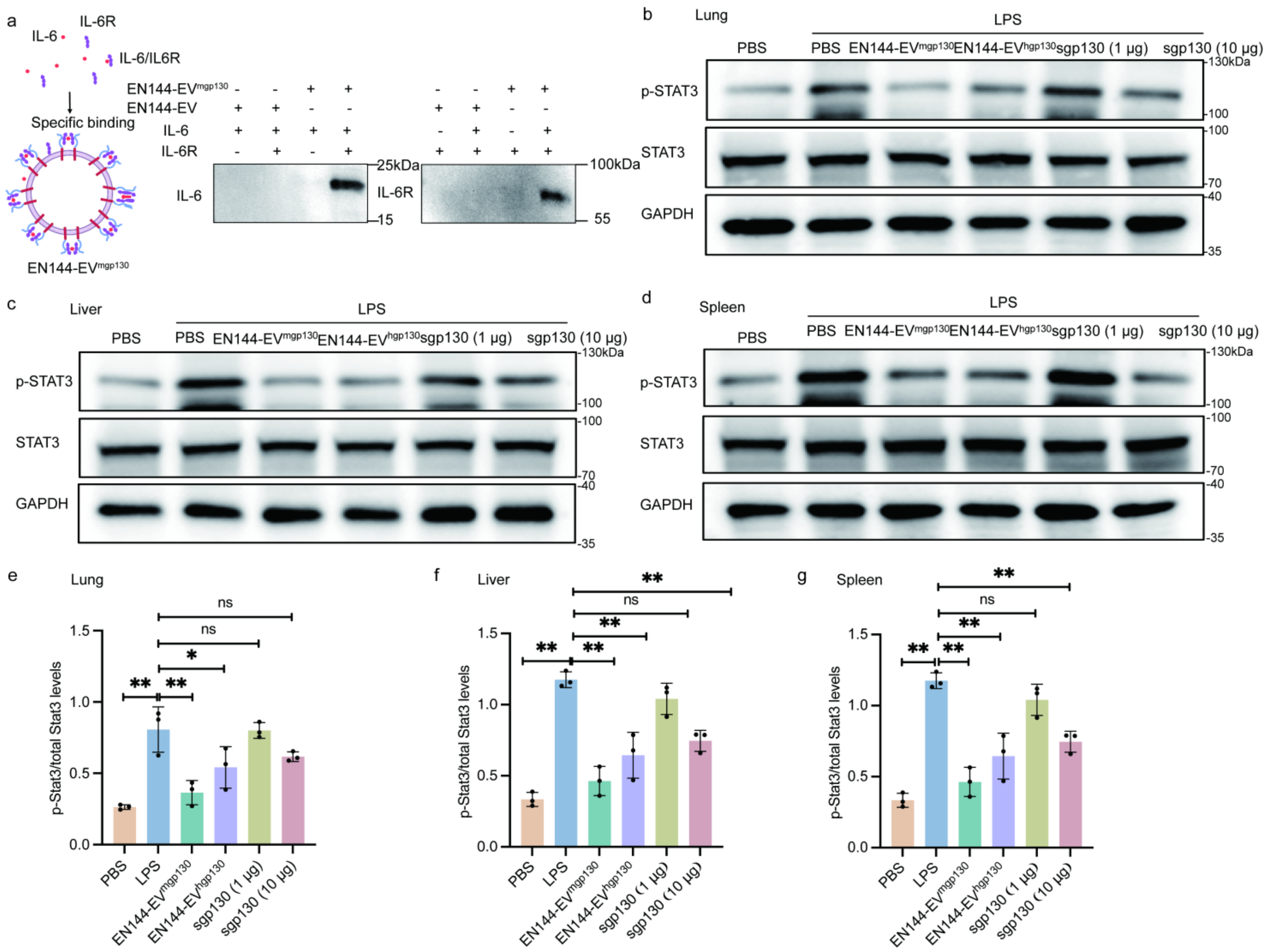

Figure S17. EN144-EV^{mgp130} targets the IL-6 trans-signaling pathway. (a) Binding capacity analysis of EN144-EV^{mgp130} or EN144-EVs to IL-6/IL-6R proteins. EN144-EV^{mgp130} or EN144-EVs (5.0×10^{10} particles) were incubated with IL-6 (50 ng), IL-6R (100 ng), or IL-6/IL-6R complex (50 ng/100 ng) under continuous rotation at 4°C for 4 h. Unbound proteins were removed by dialysis (100 kDa MWCO) against PBS for 16 h, with buffer changes every 2 h. EVs were then recovered using EVtrap magnetic beads, and samples were normalized by total protein (20 µg per lane) for western blotting. This figure was created using MedPeer (medpeer.cn). (b-d) Effects of therapeutic interventions targeting the Stat3 signaling pathway in septic mice. Experimental design: Septic mice received tail vein injections of EN144-EV^{mgp130} (1.0×10^{10} particles/mouse, 100 µL), EN144-EV^{hgp130} (1.0×10^{10} particles/mouse, 100 µL), low-dose sgp130 (1 µg/mouse, 100 µL), or high-dose sgp130 (10 µg/mouse, 100 µL). Control groups included septic mice (LPS group) and healthy mice (PBS group), both injected with an equal volume of PBS (100 µL/mouse). Lung (b), liver (c), and spleen tissues (d) were harvested post-treatment after 6 h, and expression of phosphorylated Stat3 (p-Stat3) and total Stat3 protein were analyzed by WB. (e-g) Quantitative analysis of pStat3 and Stat3 protein level in lungs (e), liver (f), and spleen (g) based on results in b-d (n = 3 each group). The data are presented as mean ± SD. The p-values (e, f, g) were analyzed by either one-way ANOVA or Brown-Forsythe ANOVA test, followed by Dunnett's or Dunnett's T3 multiple comparisons test. ns $P > 0.05$, *$P < 0.05$, **$P < 0.01$. Source data and exact p-value are provided as a Source Data file.

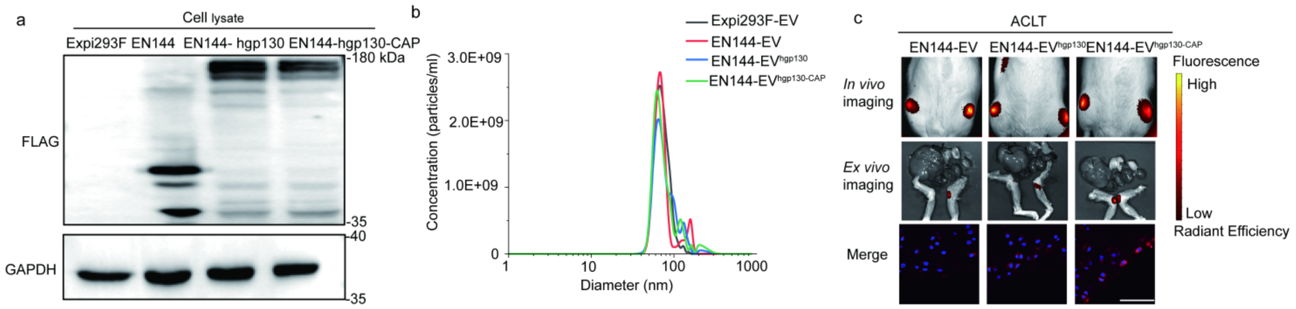

Figure S18. Construction and targeting assessment of EN144-EV$^{hgp130\text{-}CAP}$ and EN144-EV^{hgp130}. (a) The expression of FLAG-tagged protein in cell lysates of Expi293F cells transfected by EN144, EN144-hgp130, or EN144-hgp130-CAP plasmids. (b) Size distributions of EN144-EV$^{hgp130\text{-}CAP}$ and EN144-EV^{hgp130} were characterized using RPS. (c) A rat model of osteoarthritis (OA) was established based on the anterior cruciate ligament transection (ACLT) method. EV preparations (EN144-EVs, EN144-EV^{hgp130}, and EN144-EV$^{hgp130\text{-}CAP}$) of 1.0×10^{10} EV particles were injected intra-articularly into OA rats. Top panel: *In vivo* imaging of DiR-labeled EVs post-injection after 24 hours showing intra-articular distribution. Middle panel: *Ex vivo* organ imaging post-injection after at 7 days to evaluate systemic retention. Bottom panel: Fluorescence microscopy of cartilage tissue sections after injection of DiI-labeled EVs, demonstrating localized accumulation. scale bar = 50 μm. (n = 3 mice each group). Source data are provided as a Source Data file.

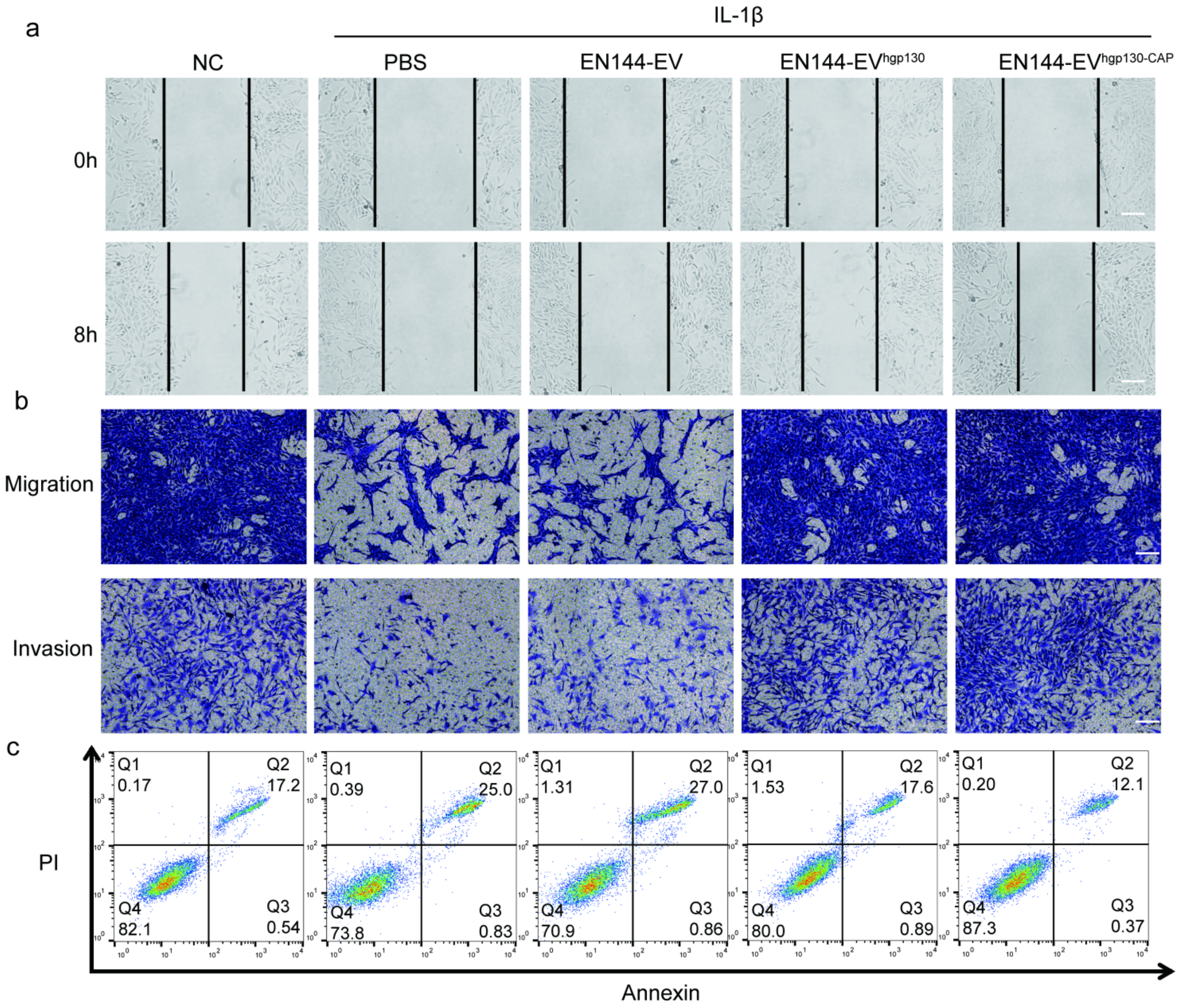

Figure S19. Effect of EN144-EV$^{hgp130-CAP}$ on osteoarthritis (OA)-like chondrocyte phenotype. (a) Rat primary chondrocytes were treated with interleukin-1β (IL-1β, 50 ng/mL) for 24 hours to establish an OA-like cellular model, followed by co-culture with equal volumes of PBS, EN144-EVs, EN144-EV^{hgp130}, or EN144-EV$^{hgp130-CAP}$ for 8 hours. Scratch wound closure distances were quantified to evaluate chondrocyte migration capacity (scale bar = 100 µm). (b) Representative images of Transwell assays showing migratory (top panel: uncoated chamber) and invasive (bottom panel: Matrigel-coated chamber) abilities of chondrocytes after 24-hour co-culture with PBS, EN144-EVs, EN144-EV^{hgp130}, or EN144-EV$^{hgp130-CAP}$ (scale bar = 100 µm). (c) Flow cytometry analysis of apoptosis ratios in chondrocytes after 24-hour co-culture with PBS, EN144-EVs, EN144-EV^{hgp130}, or EN144-EV$^{hgp130-CAP}$ using Annexin V/PI dual staining. n = 3 in each group.

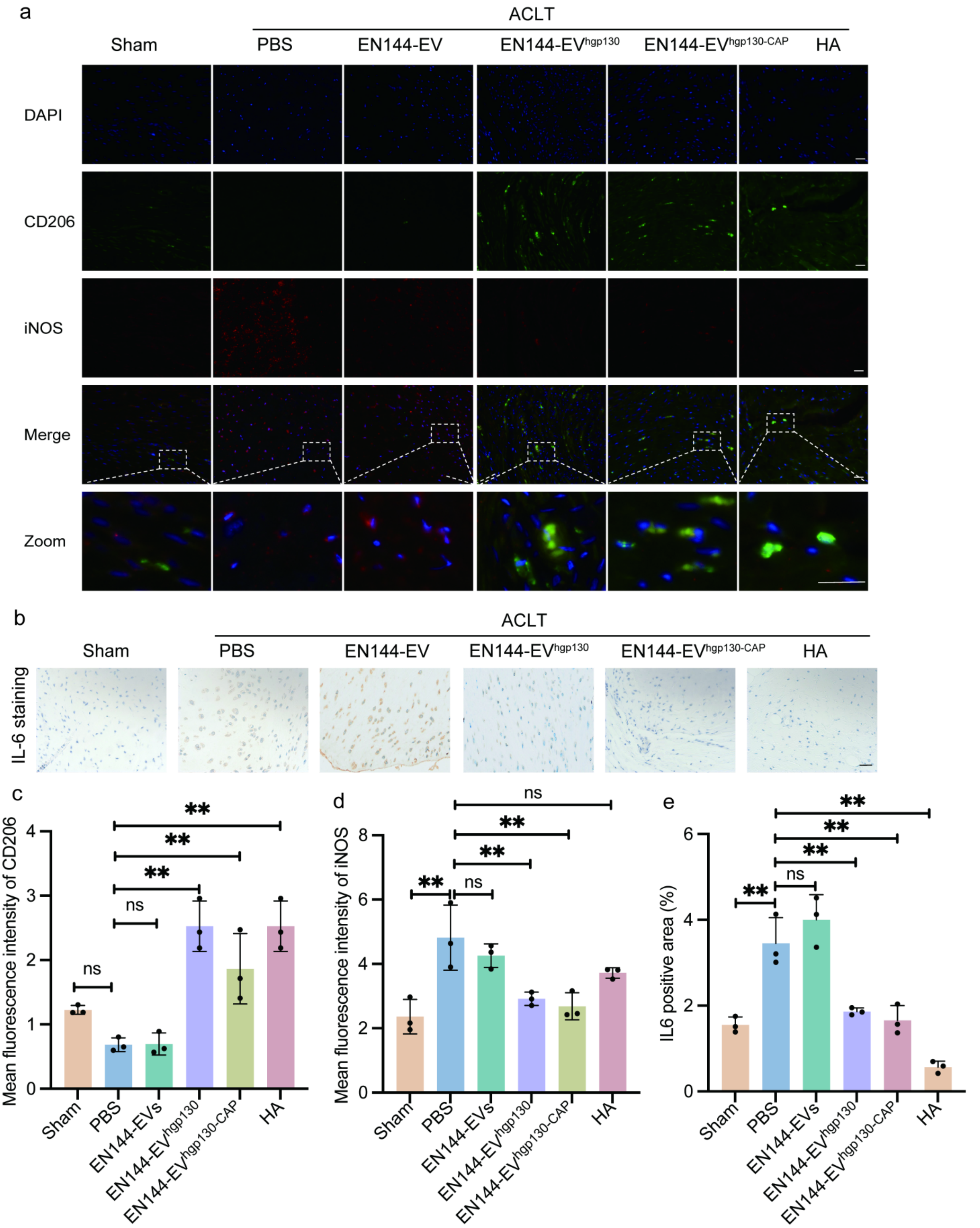

Figure S20. Modulation of synovial gene expression by EN144-EV$^{hgp130-CAP}$. A rat model of OA was established based on the ACLT method. EV preparations (EN144-EVs, EN144-EV^{hgp130}, and EN144-EV$^{hgp130-CAP}$) of 1.0×10^{10} EV particles or HA (10 μg) were injected intra-articularly into OA rats weekly for four weeks. Sham-operated controls underwent joint cavity exposure without ligament transection. The rats were sacrificed in the fifth week, and the synovial tissues were dissected for following analysis: (a) Immunofluorescence staining analysis of iNOS (red; M1 - like macrophage marker) and CD206 (green; M2 - like macrophage marker) expression in synovial tissues (scale bar = 100 μm); (b) Immunohistochemical staining of IL-6 expression in synovial tissues (scale bar =

50 μm); (c-e) Semi-quantitative analysis of protein level of CD206 (c), iNOS (d), and IL-6 (e) in synovial tissues across groups (n = 3 each group). The data are presented as mean ± SD. The p values (c, d, e) were analyzed by either one-way ANOVA, followed by Dunnett's multiple comparisons test. ns $P > 0.05$, ** $P < 0.01$. Source data and exact p-value are provided as a Source Data file.

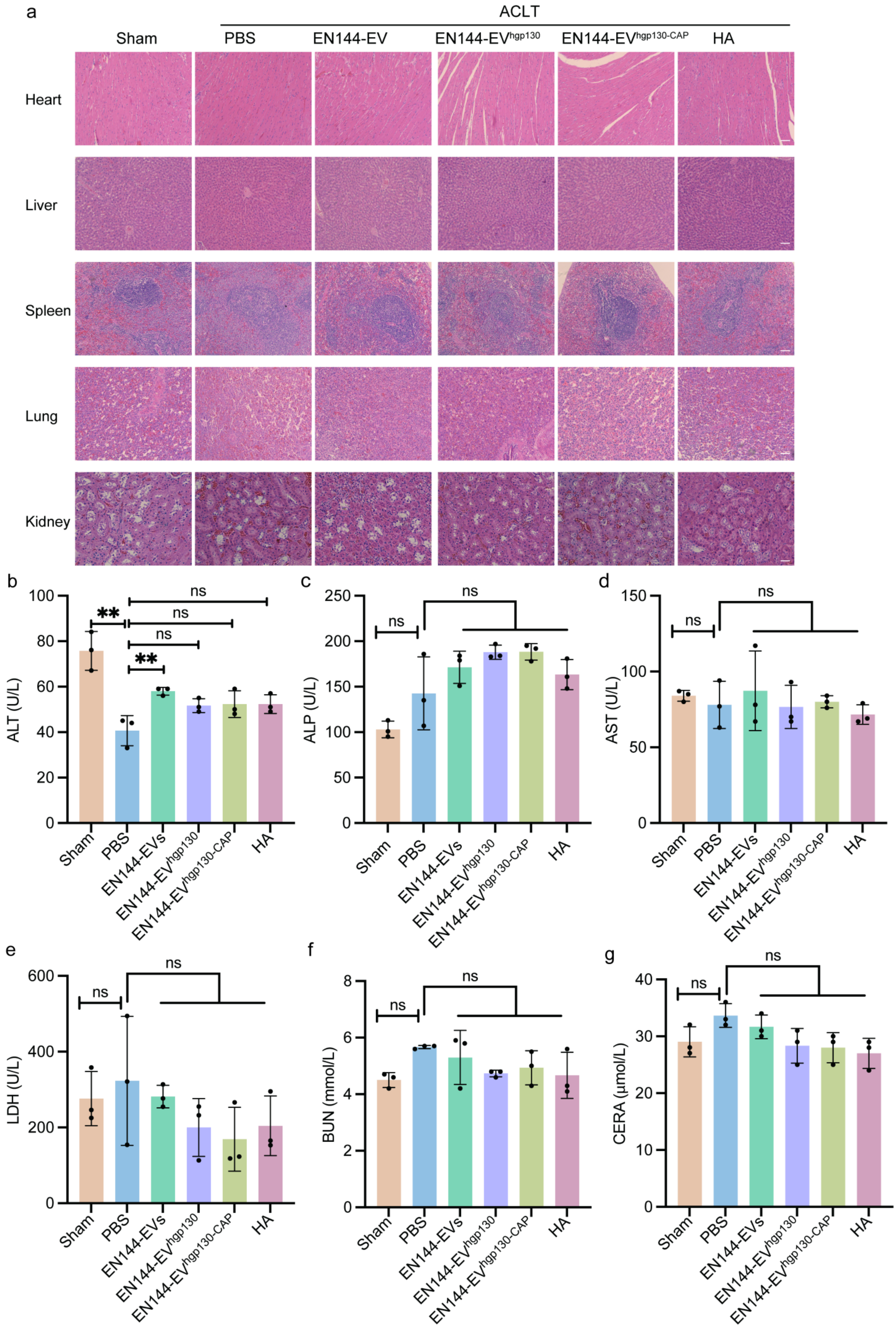
a
ACLT
Sham
PBS
EN144-EV
EN144-EV^hgp130
EN144-EV^hgp130-CAP
HA
Heart
Liver
Spleen
Lung
Kidney
b
ALT (U/L)
c
ALP (U/L)
d
AST (U/L)
e
LDH (U/L)
f
BUN (mmol/L)
g
CERA (µmol/L)
**
ns
Sham
PBS
EN144-EVs
EN144-EV^hgp130
EN144-EV^hgp130-CAP
HA

Figure S21. Assessment of toxic effects on vital organs across treatment groups. A rat model of OA was established based on the ACLT method. EV preparations (EN144-EVs, EN144-EV^{hgp130}, and EN144-$EV^{hgp130\text{-}CAP}$) of 1.0×10^{10} EV particles or HA (10 μg) were injected intra-articularly into OA rats weekly for four weeks. The rats were sacrificed in the fifth week, and heart, liver, spleen, lung, and kidney tissues were dissected for H&E staining (a), and serum samples were collected to measure levels of alanine aminotransferase (ALT, b), alkaline phosphatase (ALP, c), aspartate aminotransferase (AST, d), lactate dehydrogenase (LDH, e), blood urea nitrogen (BUN, f), and creatinine (CREA, g), systematically assessing the potential organ toxicity of treatments ($n = 3$ per group). Sham-operated controls underwent joint cavity exposure without ligament transection. Scale bar = 50 μm. One-way ANOVA accompanied by Tukey's post-hoc test was employed. Data are analyzed of three independent experiments and shown as mean ± SD. The data are presented as mean ± SD. The p values (b, c, d, e, f, g) were analyzed by either one-way ANOVA, followed by Dunnett's multiple comparisons test. ns $P > 0.05$. Source data and exact p-value are provided as a Source Data file.

Supplementary Table 1. Information on the use of antibodies.

Experiment	Antibody	Catalog	Dilution
WB	Anti-Hsp70 antibody	Abcam,ab181606	1:1000
WB	Anti-TSG101 antibody	Abcam, ab30871	1:1000
WB	Anti-CD9 antibody	Abcam, ab92726	1:1000
WB	Anti-Calnexin (CANX) antibody	Abcam, ab22595	1:1000
WB	Anti-GFP antibody	Abways, AB0047	1:1000
WB	Anti-Syntenin antibody	Abways, CY8713	1:1000
WB	Anti-Histone H3 antibody	Proteintech, 68345-1-Ig	1:1000
WB	Anti-IL-6R alpha antibody	Proteintech, 23457-1-AP	1:1000
WB	Anti-STAT3 antibody	Proteintech, 10253-2-AP	1:1000
WB	Anti-Phospho-STAT3 (Ser727) antibody	Proteintech,80199-2-RR	1:1000
IHC	Anti-MMP13 antibody	Proteintech, 18165-1-AP	1:50
IHC	Anti-Collagen Type II (COL2A1) antibody	Proteintech, 28459-1-AP	1:800
WB	Anti-GAPDH antibody	Abcam, ab9485	1:2500
WB	Anti-TNF-α antibody	Abways, AB3630	1:500
WB	Anti-IL-6 antibody	Santa Cruz biotechnology, sc-57315	1:200
WB	Anti-Cas9 antibody	Cell Signaling Technology, 14697S	1:1000
WB	Anti-FLAG antibody	Bioablab, 20221008	1:1000
IF	Anti-iNOS antibody	Proteintech, 18985-1-AP	1:3000
IF	Anti-CD206 antibody	Cell Signaling Technology, 24595	1:3000
WB	Goat Anti-Rabbit IgG H&L (HRP)	Abcam, ab205718	1:5000
WB	Goat Anti-Mouse IgG H&L (HRP)	Abcam, ab6708	1:5000

Supplementary Table 2. Amino acid sequence of the various genetic constructs used in this study.

Construct	Amino acid sequence
ENPP1	MERDGCAGGGSRGGEGGRAPREGPAGNGRDRGRSHAAEAPGDPQAAASL<br>LAPMDVGEEPLEKAARARTAKDPNTYKVLSLVLSVCVLTTILGCIFGLKPSC<br>AKEVKSCKGRCFERTFGNCRCDAACVELGNCCLDYQETCIEPEHIWTCNKF<br>RCGEKRLTRSLCACSDDCKDKGDCCINYSSVCQGEKSWVEEPCESINEPQCP<br>AGFETPPTLLFSLDGFRAEYLHTWGGLLPVISKLKKCGTYTKNMRPVYPTKT<br>FPNHYSIVTGLYPESHGIIDNKMYDPKMNASFSLKSKEKFNPEWYKGEPIWV<br>TAKYQGLKSGTFFWPGSDVEINGIFPDIYKMYNGSVPFEERILAVLQWLQLP<br>KDERPHFYTLYLEEPDSSGHSYGPVSSEVIKALQRVDGMVGMLMDGLKEL<br>NLHRCLNLILISDHGMEQGSCKKYIYLNKYLGDVKNIKVIYGPAARLRPSDV<br>PDKYYSFNYEGIARNLSCREPNQHFKPYLKHFLPKRLHFAKSDRIEPLTFYLD<br>PQWQLALNPSERKYCGSGFHGSDNVFSNMQALFVGYGPGFKHGIEADTFEN<br>IEVYNLMCDLLNLTPAPNNGTHGSLNHLLKNPVYTPKHPKEVHPLVQCPFT<br>RNPRDNLGCSCNPSILPIEDFQTQFNLTVAEEKIIKHETLPYGRPRVLQKENTI<br>CLLSQHQFMSGYSQDILMPLWTSYTVDRNDSFSTEDFSNCLYQDFRIPLSPV<br>HKCSFYKNNTKVSYGFLSPPQLNKNSSGIYSEALLTTNIVPMYQSFQVIWRY<br>FHDTLLRKYAEERNGVNVVSGPVFDFDYDGRCDSLENLRQKRRVIRNQEILI<br>PTHFFIVLTSCKDTSQTPLHCENLDTLAFILPHRTDNSESCVHGKHDSSWVEE<br>LLMLHRARITDVEHITGLSFYQQRKEPVSDILKLKTHLPTFSQED
Rab7a	TSRKKVLLKVIILGDSGVGKTSLMNQYVNKKFSNQYKATIGADFLTKEVMV<br>DDRLVTMQIWDTAGQERFQSLGVAFYRGADCCVLVFDVTAPNTFKTLDSW<br>RDEFLIQASPRDPENFPFVVLGNKIDLENRQVATKRAQAWCYSKNNIPYFET<br>SAKEAINVEQAFQTIARNALKQETEVELYNEFPEPIKLDKNDRAKASAESCS<br>C
STX7	SYTPGVGGDPAQLAQRISSNIQKITQCSVEIQRTLNQLGTPQDSPELRQQLQQ<br>KQQYTNQLAKETDKYIKEFGSLPTTPSEQRQRKIQKDRLVAEFTTSLTNFQK<br>VQRQAAEREKEFVARVRASSRVSGSFPEDSSKERNLVSWESQTQPQVQVQD<br>EEITEDDLRLIHERESSIRQLEADIMDINEIFKDLGMMIHEQGDVIDSIEANVE<br>NAEVHVQQANQQLSRAADYQRKSRKTLCIIILILVIGVAIISLIIWGLNH
CXADR	LSITTPEEMIEKAKGETAYLPCKFTLSPEDQGPLDIEWLISPADNQKVDQVIIL<br>YSGDKIYDDYYPDLKGRVHFTSNDLKSGDASINVTNLQLSDIGTYQCKVKK<br>APGVANKKIHLVVLVKPSGARCYVDGSEEIGSDFKIKCEPKEGSLPLQYEWQ<br>KLSDSQKMPTSWLAEMTSSVISVKNASSEYSGTYSCTVRNRVGSDQCLLRL<br>NVVPPSNKAGLIAGAIIGTLLALALIGLIIFCCRKKRREEKYEKEVHHDIREDV<br>PPPKSRTSTARSYIGSNHSSLGSMSPSNMEGYSKTQYNQVPSEDFERTPQSPT<br>LPPAKVAAPNLSRMGAIPVMIPAQSKDGSIV
EPCAM	QEECVCENYKLAVNCFVNNNRQCQCTSVGAQNTVICSKLAAKCLVMKAE<br>MNGSKLGRRAKPEGALQNNDGLYDPDCDESGLFKAKQCNGTSMCWCVNT<br>AGVRRTDKDTEITCSERVRTYWIIIELKHKAREKPYDSKSLRTALQKEITTRY<br>QLDPKFITSILYENNVITIDLVQNSSQKTQNDVDIADVAYYFEKDVKGESLFH<br>SKKMDLTVNGEQLDLDPGQTLIYYVDEKAPEFSMQGLKAGVIAVIVVVVIA<br>VVAGIVVLVISRKKRMAKYEKAEIKEMGEMHRELNA
TFRC	MMDQARSAFSNLFGGEPLSYTRFSLARQVDGDNSHVEMKLAVDEEENADN<br>NTKANVTKPKRCSGSICYGTIAVIVFFLIGFMIGYLGYCKGVEPKTECERLAG<br>TESPVREEPGEDFPAARRLYWDDLKRKLSEKLDSTDFTGTIKLLNENSYVPR

	EAGSQKDENLALYVENQFREFKLSKVWRDQHFVKIQVKDSAQNSVIIVDKNGRLVYLVENPGGYVAYSKAATVTGKLVHANFGTKKDFEDLYTPVNGSIVIVRAGKITFAEKVANAESLNAIGVLIYMDQTKFPIVNAELSFFGHAHLGTGDPYTPGFPSFNHTQFPPSRSSGLPNIPVQTISRAAAEKLFGNMEGDCPSDWKTDSTCRMVTSESKNVKLTVSNVLKEIKILNIFGVIKGFVEPDHYVVVGAQRDAWGPGAAKSGVGTALLLKLAQMFSDMVLKDGFQPSRSIIFASWSAGDFGSVGATEWLEGYLSSLHLKAFTYINLDKAVLGTSNFKVSASPLLYTLIEKTMQNVKHPVTGQFLYQDSNWASKVEKLTLDNAAFPFLAYSGIPAVSFCFCEDTDYPYLGTTMDTYKELIERIPELNKVARAAAEVAGQFVIKLTHDVELNLDYERYNSQLLSFVRDLNQYRADIKEMGLSLQWLYSARGDFFRATSRLTTDFGNAEKTDRFVMKKLNDRVMRVEYHFLSPYVSPKESPFRHVFWGSGSHTLPALLENLKLRKQNNGAFNETLFRNQLALATWTIQGAANALSGDVWDIDNEF
TFRC-81	MMDQARSAFSNLFGGEPLSYTRFSLARQVDGDNSHVEMKLAVDEEENADNNTKANVTKPKRCSGSICYGTIAVIVFFLIGC
VTA1	AALAPLPPLPAQFKSIQHHLRTAQEHDKRDPVVAYYCRLYAMQTGMKIDSKTPECRKFLSKLMDQLEALKKQLGDNEAITQEIVGCAHLENYALKMFLYADNEDRAGRFHKNMIKSFYTASLLIDVITVFGELTDENVKHRKYARWKATYIHNCLKNGETPQAGPVGIEEDNDIEENEDAGAASLPTQPTQPSSSSTYDPSNMPSGNYTGIQIPPGAHAPANTPAEVPHSTGVASNTIQPTPQTIPAIDPALFNTISQGDVRLTPEDFARAQKYCKYAGSALQYEDVSTAVQNLQKALKLLTTGRE
Syntenin-1	IKLWTNRDLHIIVMVKNPAKMSLYPSLEDLKVDKVIQAQTAFSANPANPAILSEASAPIPHDGNLYPRLYPELSQYMGLSLNEEEIRANVAVVSGAPLQGLVARPSSINYMVAPVTGNDVGIRRAEIKQGIREVILCKDQDGKIGLRLKSIDNGIFVQLVQANSPASLVGLRFGDQVLQINGENCAGWSSDKAHKVLKQAFGEKITMTIRDRPFERTITMHKDSTGHVGFIFKNGKITSIVKDSSAARNGLLTEHNICEINGQNVIGLKDSQIADILSTSGTVVTITIMPAFIFEHIIKRMAPSIMKSLMDHTIPEV
SNAP23	DNLSSEEIQQRAHQITDESLESTRRILGLAIESQDAGIKTITMLDEQKEQLNRIEEGLDQINKDMRETEKTLTELNKCCGLCVCPCNRTKNFESGKAYKTTWGDGGENSPCNVVSKQPGPVTNGQLQQPTTGAASGGYIKRITNDAREDEMEENLTQVGSILGNLKDMALNIGNEIDAQNPQIKRITDKADTNRDRIDIANARAKKLIDS
AT1A1	DELKKEVSMDDHKLSLDELHRKYGTDLSRGLTSARAAEILARDGPNALTPPPTTPEWIKFCRQLFGGFSMLLWIGAILCFLAYSIQAATEEEPQNDNLYLGVVLS
STX4	MRDRTHELRQGDDSSDEEDKERVALVVHPGTARLGSPDEEFFHKVRTIRQTIVKLGNKVQELEKQQVTILATPLPEESMKQELQNLRDEIKQLGREIRLQLKAIEPQKEEADENYNSVNTRMRKTQHGVLSQQFVELINKCNSMQSEYREKNVERIRRQLKITNAGMVSDEELEQMLDSGQSEVFVSNILKDTQVTRQALNEISARHSEIQQLERSIRELHDIFTFLATEVEMQGEMINRIEKNILSSADYVERGQEHVKTALENQKKARKKKVLIAICVSITVVLLAVIIGVTVVG
PDL1	FTVTVPKDLYVVEYGSNMTIECKFPVEKQLDLAALIVYWEMEDKNIIQFVHGEEDLKVQHSSYRQRARLLKDQLSLGNAALQITDVKLQDAGVYRCMISYGGADYKRITVKVNAPYNKINQRILVVDPVTSEHELTCQAEGYPKAEVIWTSSDHQVLSGKTTTTNSKREEKLFNVTSTLRINTTTNEIFYCTFRRLDPEENHTAEL

	VIPELPLAHPPNERTHLVILGAILLCLGVALTFIFRLRKGRMMDVKKCGIQDT<br>NSKKQSDTHLEET
VAMP2	MSATAATAPPAAPAGEGGPPAPPPNLTSNRRLQQTQAQVDEVVDIMRVNV<br>DKVLERDQKLSELDDRADALQAGASQFETSAAKLKRKYWWKNLKMMIIL<br>GVICAIILIIIIVYFST
CD55	DCGLPPDVPNAQPALEGRTSFPEDTVITYKCEESFVKIPGEKDSVICLKGSQW<br>SDIEEFCNRSCEVPTRLNSASLKQPYITQNYFPVGTVVEYECRPGYRREPSLS<br>PKLTCLQNLKWSTAVEFCKKKSCPNPGEIRNGQIDVPGGILFGATISFSCNTG<br>YKLFGSTSSFCLISGSSVQWSDPLPECREIYCPAPPQIDNGIIQGERDHYGYRQ<br>SVTYACNKGFTMIGEHSIYCTVNNDEGEWSGPPPECRGKSLTSKVPPTVQKP<br>TTVNVPTTEVSPTSQKTTTKTTTPNAQATRSTPVSRTTKHFHETTPNKGSGT<br>TSGTTRLLSGHTCFTLTGLLGTLVTMGLLT
IST1	LGSGFKAERLRVNLRLVINRLKLLEKKKTELAQKARKEIADYLAAGKDERA<br>RIRVEHIIREDYLVEAMEILELYCDLLLARFGLIQSMKELDSGLAESVSTLIW<br>AAPRLQSEVAELKIVADQLCAKYSKEYGKLCRTNQIGTVNDRLMHKLSVEA<br>PPKILVERYLIEIAKNYNVPYEPDSVVMAEAPPGVETDLIDVGFTDDVKKGG<br>PGRGGSGGFTAPVGGPDGTVPMPMPMPMPSANTPFSYPLPKGPSDFNGLPM<br>GTYQAFPNIHPPQIPATPPSYESVDDINADKNISSAQIVGPGPKPEASAKLPSR<br>PADNYDNFVLPELPSVPDTLPTASAGASTSASEDIDFDDLSRRFEELKKKT
PTGFRN	VPTATLVRVVGTELVIPCNVSDYDGPSEQNFDWSFSSLGSSFVELASTWEVG<br>FPAQLYQERLQRGEILLRRTANDAVELHIKNVQPSDQGHYKCSTPSTDATV<br>QGNYEDTVQVKVLADSLHVGPSARPPPSLSLREGEPFELRCTAASASPLHTH<br>LALLWEVHRGPARRSVLALTHEGRFHPGLGYEQRYHSGDVRLDTVGSDAY<br>RLSVSRALSADQGSYRCIVSEWIAEQGNWQEIQEKAVEVATVVIQPSVLRA<br>AVPKNVSVAEGKELDLTCNITTDRADDVRPEVTWSFSRMPDSTLPGSRVLA<br>RLDRDSLVHSSPHVALSHVDARSYHLLVRDVSKENSGYYYCHVSLWAPGH<br>NRSWHKVAEAVSSPAGVGVTWLEPDYQVYLNASKVPGFADDPTELACRV<br>VDTKSGEANVRFTVSWYYRMNRRSDNVVTSELLAVMDGDWTLKYGERSK<br>QRAQDGDFIFSKEHTDTFNFRIQRTTEEDRGNYYCVVSAWTKQRNNSWVKS<br>KDVFSKPVNIFWALEDSVLVVKARQPKPFFAAGNTFEMTCKVSSKNIKSPR<br>YSVLIMAEKPVGDLSSPNETKYIISLDQDSVVKLENWTDASRVDGVVLEKV<br>QEDEFRYRMYQTQVSDAGLYRCMVTAWSPVRGSLWREAATSLSNPIEIDFQ<br>TSGPIFNASVHSDTPSVIRGDLIKLFCIITVEGAALDPDDMAFDVSWFAVHSF<br>GLDKAPVLLSSLDRKGIVTTSRRDWKSDLSLERVSVLEFLLQVHGSEDQDFG<br>NYYCSVTPWVKSPTGSWQKEAEIHSKPVFITVKMDVLNAFKYPLLIGVGLS<br>TVIGLLSCLIGYCSSHWCCKKEVQETRRERRRLMSMEMD
Lamp2b	LELNLTDSENATCLYAKWQMNFTVRYETTNKTYKTVTISDHGTVTYNGSIC<br>GDDQNGPKIAVQFGPGFSWIANFTKAASTYSIDSVSFSYNTGDNTTFPDAED<br>KGILTVDELLAIRIPLNDLFRCNSLSTLEKNDVVQHYWDVLVQAFVQNGTV<br>STNEFLCDKDKTSTVAPTIHTTVPSPTTTPTPKEKPEAGTYSVNNGNDTCLLA<br>TMGLQLNITQDKVASVININPNTTHSTGSCRSHTALLRLNSSTIKYLDFVFAV<br>KNENRFYLKEVNISMYLVNGSVFSIANNNLSYWDAPLGSSYMCNKEQTVSV<br>SGAFQINTFDLRVQPFNVTQGKYSTAQECSLDDDTILIPIIVGAGLSGLIIVIVI<br>AYVIGRRKSYAGYQTL
EN52	MERDGCAGGGSRGGEGGRAPREGPAGNGRDRGRSHAAEAPGDPQAAASL<br>LAP

EN144	MERDGCAGGGSRGGEGGRAPREGPAGNGRDRGRSHAAEAPGDPQAAASL<br>LAPMDVGEEPLEKAARARTAKDPNTYKVLSLVLSVCVLTTILGCIFGLKPSC<br>AKEVKSCKGRCFERTFGNCRCDAACVELGNCCLDYQETCIEPE
EN190	MERDGCAGGGSRGGEGGRAPREGPAGNGRDRGRSHAAEAPGDPQAAASL<br>LAPMDVGEEPLEKAARARTAKDPNTYKVLSLVLSVCVLTTILGCIFGLKPSC<br>AKEVKSCKGRCFERTFGNCRCDAACVELGNCCLDYQETCIEPEHIWTCNKF<br>RCGEKRLTRSLCACSDDCKDKGDCCINYSSVCQGEKSW
EN596	MERDGCAGGGSRGGEGGRAPREGPAGNGRDRGRSHAAEAPGDPQAAASL<br>LAPMDVGEEPLEKAARARTAKDPNTYKVLSLVLSVCVLTTILGCIFGLKPSC<br>AKEVKSCKGRCFERTFGNCRCDAACVELGNCCLDYQETCIEPEHIWTCNKF<br>RCGEKRLTRSLCACSDDCKDKGDCCINYSSVCQGEKSWVEEPCESINEPQCP<br>AGFETPPTLLFSLDGFRAEYLHTWGGLLPVISKLKKCGTYTKNMRPVYPTKT<br>FPNHYSIVTGLYPESHGIIDNKMYDPKMNASFSLKSKEKFNPEWYKGEPIWV<br>TAKYQGLKSGTFFWPGSDVEINGIFPDIYKMYNGSVPFEERILAVLQWLQLP<br>KDERPHFYTLYLEEPDSSGHSYGPVSSEVIKALQRVDGMVGMLMDGLKEL<br>NLHRCLNLILISDHGMEQGSCKKYIYLNKYLGDVKNIKVIYGPAARLRPSDV<br>PDKYYSFNYEGIARNLSCREPNQHFKPYLKHFLPKRLHFAKSDRIEPLTFYLD<br>PQWQLALNPSERKYCGSGFHGSDNVFSNMQALFVGYGPGFKHGIEADTFEN<br>IEVYNLMCDLLNLTPAPNNGTHGSLNHLLK
hTNFR1	MGLSTVPDLLLPLVLLELLVGIYPSGVIGLVPHLGDREKRDSVCPQGKYIHP<br>QNNSICCTKCHKGTYLYNDCPGPGQDTDCRECESGSFTASENHLRHCLSCS<br>KCRKEMGQVEISSCTVDRDTVCGCRKNQYRHYWSENLFQCFNCSLCLNGT<br>VHLSCQEKQNTVCTCHAGFFLRENECVSCSNCKKSLECTKLCLPQIENVKGT<br>EDSGTTVLLPLVIFFGLCLLSLLFIGLMYRYQRWKSKLYSIVCGKSTPEKEGE<br>LEGTTTKPLAPNPSFSPTPGFTPTLGFSPVPSSTFTSSSTYTPGDCPNFAAPRRE<br>VAPPYQGADPILATALASDPIPNGGGGSGRVILEGGHHHHHH
mgp130	MSAPRIWLAQALLFFLTTESIGQLLEPCGYIYPEFPVVQRGSNFTAICVLKEA<br>CLQHYYVNASYIVWKTNHAAVPREQVTVINRTTSSVTFTDVVLPSVQLTCN<br>ILSFGQIEQNVYGVTMLSGFPPDKPTNLTCIVNEGKNMLCQWDPGRETYLET<br>NYTLKSEWATEKFPDCQSKHGTSCMVSYMPTYYVNIEVWVEAENALGKVS<br>SESINFDPVDKVKPTPPYNLSVTNSEELSSILKLSWVSSGLGGLLDLKSDIQY<br>RTKDASTWIQVPLEDTMSPRTSFTVQDLKPFTEYVFRIRSIKDSGKGYWSDW<br>SEEASGTTYEDRPSRPPSFWYKTNPSHGQEYRSVRLIWKALPLSEANGKILD<br>YEVILTQSKSVSQTYTVTGTELTVNLTNDRYVASLAARNKVGKSAAAVLTI<br>PSPHVTAAYSVVNLKAFPKDNLLWVEWTPPPKPVSKYILEWCVLSENAPCV<br>EDWQQEDATVNRTHLRGRLLESKCYQITVTPVFATGPGGSESLKAYLKQAA<br>PARGPTVRTKKVGKNEAVLAWDQIPVDDQNGFIRNYSISYRTSVGKEMVV<br>HVDSSHTEYTLSSLSSDTLYMVRMAAYTDEGGKDGPEFTFTTPKFAQGEIEA<br>IVVPVCLAFLLTTLLGVLFCFNKRDLIKKHIWPNVPDPSKSHIAQWSPHTPPR<br>HNFNSKDQGGGGSHHHHHH
hgp130	MLTLQTWLVQALFIFLTTESTGELLDPCGYISPESPVVQLHSNFTAVCVLKE<br>KCMDYFHVNANYIVWKTNHFTIPKEQYTIINRTASSVTFTDIASLNIQLTCNI<br>LTFGQLEQNVYGITIISGLPPEKPKNLSCIVNEGKKMRCEWDGGRETHLETN<br>FTLKSEWATHKFADCKAKRDTPTSCTVDYSTVYFVNIEVWVEAENALGKV<br>TSDHINFDPVYKVKPNPPHNLSVINSEELSSILKLTWTNPSIKSVIILKYNIQYR<br>TKDASTWSQIPPEDTASTRSSFTVQDLKPFTEYVFRIRCMKEDGKGYWSDW

SEEASGITYEDRPSKAPSFWYKIDPSHTQGYRTVQLVWKTLPPFEANGKILD
YEVTLTRWKSHLQNYTVNATKLTVNLTNDRYLATLTVRNLVGKSDAAVLT
IPACDFQATHPVMDLKAFPKDNMLWVEWTTPRESVKKYILEWCVLSDKAP
CITDWQQEDGTVHRTYLRGNLAESKCYLITVTPVYADGPGSPESIKAYLKQ
APPSKGPTVRTKKVGKNEAVLEWDQLPVDVQNGFIRNYTIFYRTIIGNETAV
NVDSSHTEYTLSSLTSDTLYMVRMAAYTDEGGKDGPEFTFTTPKFAQGEIE
AIVVPVCLAFLLTTLLGVLFCFNKRDLIKKHIWPNVPDPSKSHIAQWSPHTPP
RHNFNSKDQMYSDGNFTDVSVVEIEANDKKPFPEDLKSLDLFKKEKINTEG
HSSGIGGSSCMSSSRPSISSSDENESSQNTSSTVQYSTVVHSGYRHQVPSVQV
FSRSESTQPLLDSEERPEDLQLVDHVDGGDGILPRQQYFKQNCSQHESSPDIS
HFERSKQVSSVNEEDFVRLKQQISDHISQSCGSGQMKMFQEVSAADAFGPG
TEGQVERFETVGMEAATDEGMPKSYLPQTVRQGGYMPQGGGGSHHHHHH

Supplementary Table 3. Sequence information of primers.

Primers	species	sequence（5'-3'）
EGFP-F	foreign gene	CAGTGCTTCAGCCGCTACCC
EGFP-R	foreign gene	TTCACCTTGATGCCGTTCTT
Cas9-F	foreign gene	CTGCCAGACTGAGCAAGAG
Cas9-R	foreign gene	GGTCGTCGTCGTAGGTGT
sgRNA1-F	foreign gene	CAGAATATCACACAGCGTTT
sgRNA1-R	foreign gene	GTTGATAACGGACTAGCCTT
IL-6-F	Mouse	TCGGAGGCTTAATTACACATGTTCT
IL-6-R	Mouse	GCATCATCGTTGTTCATACAATCA
TNF-α-F	Mouse	CCACCACGCTCTTCTGTCTAC
TNF-α-R	Mouse	AGGGTCTGGGCCATAGAACT
GAPDH-F	Human	GATCATCAGCAATGCCTCCT
GAPDH-R	Human	TGTGGTCATGAGTCCTTCCA
GAPDH-F	Mouse	CATCAAGAAGGTGGTGAAGC
GAPDH-R	Mouse	CCTGTTGCTGTAGCCGTATT

Supplementary Table 4. Expression of candidate scaffold proteins in Expi293F-EVs.

Protein	Method 1	Method 2	Method 3
AT1A1	526710708	138277052.25	1026389218
CD55	32024020.5	19512350.94	94431336.75
CXADR	42456913.44	10715861.53	77720049.28
ENPP1	15286231	1631481.97	54971684.78
EPCAM	12475875.5	1886664.06	16084135.63
IST1	38748758.16	10643372.14	21546999.88
PTGFRN	399616063.4	161018892.02	85404108.38
Rab7a	129334979.3	36462442.73	264679295.1
Syntenin-1	142595787.1	52954778.23	119848406.6
SNAP23	52726180.56	11516722.03	89221599.81
STX4	19893865.31	5807356.55	54896545.33
STX7	28163980.13	11506500.01	20485312.13
TFRC	213339809	40406221.80	14986063.34
VAMP2	49801861.69	15444213.56	50328112
VTA1	60874827.94	13773779.69	47931117.38

Supplementary Table 5. Efficiency of Indel Generation calculated with TIDE.

Gene	Editing efficacy	R^2
Stop-dsRed (Rep1)	13.6%	0.98
Stop-dsRed (Rep2)	5.7%	0.96
Stop-dsRed (Rep3)	3.1%	0.97
