# Supplementary material for: Extracellular vesicle engineering using a small scaffold protein
Source: Nat Commun. 2026 Mar 10;17:3726. doi: 10.1038/s41467-026-70451-x (PMC13102933; doi:10.1038/s41467-026-70451-x)
Supplement: Supplementary file 2 — Reporting Summary [file 41467_2026_70451_MOESM2_ESM.pdf]

Corresponding author(s): Shizhi Wang, Evan Yi-Wen Yu, Wei qin Li, Jiang Xia, Hao Zhang

Last updated by author(s): Feb 9, 2026

# Reporting Summary

Nature Portfolio wishes to improve the reproducibility of the work that we publish. This form provides structure for consistency and transparency in reporting. For further information on Nature Portfolio policies, see our Editorial Policies and the Editorial Policy Checklist.

## Statistics

For all statistical analyses, confirm that the following items are present in the figure legend, table legend, main text, or Methods section.

| n/a | Confirmed | |
|---|---|---|
| ☐ | ☒ | The exact sample size ($n$) for each experimental group/condition, given as a discrete number and unit of measurement |
| ☐ | ☒ | A statement on whether measurements were taken from distinct samples or whether the same sample was measured repeatedly |
| ☐ | ☒ | The statistical test(s) used AND whether they are one- or two-sided<br>*Only common tests should be described solely by name; describe more complex techniques in the Methods section.* |
| ☒ | ☐ | A description of all covariates tested |
| ☒ | ☐ | A description of any assumptions or corrections, such as tests of normality and adjustment for multiple comparisons |
| ☐ | ☒ | A full description of the statistical parameters including central tendency (e.g. means) or other basic estimates (e.g. regression coefficient) AND variation (e.g. standard deviation) or associated estimates of uncertainty (e.g. confidence intervals) |
| ☒ | ☐ | For null hypothesis testing, the test statistic (e.g. $F$, $t$, $r$) with confidence intervals, effect sizes, degrees of freedom and $P$ value noted<br>*Give P values as exact values whenever suitable.* |
| ☒ | ☐ | For Bayesian analysis, information on the choice of priors and Markov chain Monte Carlo settings |
| ☒ | ☐ | For hierarchical and complex designs, identification of the appropriate level for tests and full reporting of outcomes |
| ☒ | ☐ | Estimates of effect sizes (e.g. Cohen's $d$, Pearson's $r$), indicating how they were calculated |

*Our web collection on statistics for biologists contains articles on many of the points above.*

## Software and code

Policy information about availability of computer code

| | |
|---|---|
| Data collection | 1. Flow cytometric data were collected using FACS Calibur (BD Biosciences).<br>2. Western blotting Assay data were collected using Tanon-4160.<br>3. Fluorescence images were acquired using a Olympus FV1000 confocal microscope.<br>4. Mass Spectrometry was performed on a Thermo Fisher QE-HFX mass spectrometer.<br>5. In vivo distribution imaging was obtained using IVIS Lumina III (PerkinElmer).<br>6. The extracellular vesicles were analyzed using a NanoCoulter counter (Resun Technology, China).<br>7. The transmission electron microscopy (TEM) images were taken with a HITACHI transmission electron microscope.<br>8. Immunohistochemistry imaging was obtained using Olympus VS200. |
| Data analysis | 1. Statistical analysis was performed with GraphPad Prism (version 8.0).<br>2. FACS data was analyzed with FlowJo software (version 10.8).<br>3. The size and concentration of extracellular vesicles were analyzed by Nanocoulter software (version 3.2).<br>4. Western blot bands and immunohistochemistry images were analyzed by Image J software (version 1.8.0) |

For manuscripts utilizing custom algorithms or software that are central to the research but not yet described in published literature, software must be made available to editors and reviewers. We strongly encourage code deposition in a community repository (e.g. GitHub). See the Nature Portfolio guidelines for submitting code & software for further information.

## Data

Policy information about availability of data

All manuscripts must include a data availability statement. This statement should provide the following information, where applicable:

- Accession codes, unique identifiers, or web links for publicly available datasets
- A description of any restrictions on data availability
- For clinical datasets or third party data, please ensure that the statement adheres to our policy

The mass spectrometry proteomics data have been deposited to the ProteomeXchange Consortium via the PRIDE partner repository with the dataset identifier PXD057965 Due to the large size, raw microscopy and immunohistochemistry data are not included in the source data but are available upon request. Plasmid descriptions and amino acid sequence used in each experiment can be found in Supplementary Data 2. The authors declare that all other data that support the findings of this study are available within the paper, its supplementary information, or Source Data file.

## Research involving human participants, their data, or biological material

Policy information about studies with human participants or human data. See also policy information about sex, gender (identity/presentation), and sexual orientation and race, ethnicity and racism.

| | |
|---|---|
| Reporting on sex and gender | N/A |
| Reporting on race, ethnicity, or other socially relevant groupings | N/A |
| Population characteristics | N/A |
| Recruitment | N/A |
| Ethics oversight | N/A |

Note that full information on the approval of the study protocol must also be provided in the manuscript.

# Field-specific reporting

Please select the one below that is the best fit for your research. If you are not sure, read the appropriate sections before making your selection.

☒ Life sciences ☐ Behavioural & social sciences ☐ Ecological, evolutionary & environmental sciences

For a reference copy of the document with all sections, see nature.com/documents/nr-reporting-summary-flat.pdf

# Life sciences study design

All studies must disclose on these points even when the disclosure is negative.

| | |
|---|---|
| Sample size | For in vivo studies, n=3 - 5 mice per group is sufficient to detect meaningful biological differences with good reproducibility. For immunohistochemistry assays, we typically performed at least three independent experiment repeats to allow statistical analysis and robust conclusions to be drawn. Protein blot, vesicle counts and sizes, were conducted at least two independent experiments. We determined the sample size to be sufficient based on literature (PMID: 38297121, 38965227 and 37552983). |
| Data exclusions | No data were excluded from this study. |
| Replication | In almost all cases, experiments were replicated at least twice in a consistent manner to verify results and conclusions. Proteomic analysis using each method in Figure 1A extracted extracellular vesicles, with one analysis identifying common proteins. A single mass spectrometry detection on each group of vesicles in Figure 1C sufficiently confirmed the findings in Figure S3. |
| Randomization | Animals used were randomly assigned to each treatment group. No particular randomization method was used for other experiments. Randomization is not applicable for most standard cell culture based assays and in vitro experiments. But, the same number of cells were plated for each treatment condition and same amount of extracellular vesicles were used for each experimental condition. |
| Blinding | Blinding was not implemented in this study. The authors concurred that, given the quantitative nature of the techniques employed, such as flow cytometry or at least semi-quantitative methods (e.g., protein blot, immunohistochemistry), the outcome was unlikely to be influenced by the absence of blinding. |

# Behavioural & social sciences study design

All studies must disclose on these points even when the disclosure is negative.

Study description

Research sample

Sampling strategy

Data collection

Timing

Data exclusions

Non-participation

Randomization

# Ecological, evolutionary & environmental sciences study design

All studies must disclose on these points even when the disclosure is negative.

Study description

Research sample

Sampling strategy

Data collection

Timing and spatial scale

Data exclusions

Reproducibility

Randomization

Blinding

Did the study involve field work? ☐ Yes ☐ No

## Field work, collection and transport

Field conditions

Location

Access & import/export

Disturbance

# Reporting for specific materials, systems and methods

We require information from authors about some types of materials, experimental systems and methods used in many studies. Here, indicate whether each material, system or method listed is relevant to your study. If you are not sure if a list item applies to your research, read the appropriate section before selecting a response.

## Materials & experimental systems

| n/a | Involved in the study |
|---|---|
| ☐ | ☒ Antibodies |
| ☐ | ☒ Eukaryotic cell lines |
| ☒ | ☐ Palaeontology and archaeology |
| ☐ | ☒ Animals and other organisms |
| ☒ | ☐ Clinical data |
| ☒ | ☐ Dual use research of concern |
| ☒ | ☐ Plants |

## Methods

| n/a | Involved in the study |
|---|---|
| ☒ | ☐ ChIP-seq |
| ☐ | ☒ Flow cytometry |
| ☒ | ☐ MRI-based neuroimaging |

# Antibodies

Antibodies used

See Supplementary Table1 for complete information on antibodies used to include application, dilution, and catalog.

Validation

Antibodies validated by manufacturer: Anti-Hsp70 (Abcam, ab181606, https://www.abcam.cn/products/primary-antibodies/hsp70-antibody-epr16892-ab181606.html, used in 87 publications), Anti-TSG101 (Abcam, ab30871, https://www.abcam.cn/products/primary-antibodies/tsg101-antibody-ab30871.html, used in 191 publications), Anti-CD9 (Abcam, ab92726, detected protein at correct size in this study), Anti-Calnexin (Abcam, ab22595, https://www.abcam.cn/products/primary-antibodies/calnexin-antibody-er-marker-ab22595.html, used in 482 publications), Anti-GAPDH (Abcam, ab9485, https://www.abcam.cn/products/primary-antibodies/gapdh-antibody-loading-control-ab9485.html, used in 3283 publications), Anti-Cas9 (Cell Signaling Technology, 14697S, https://www.cellsignal.cn/products/primary-antibodies/cas9-s-pyogenes-7a9-3a3-mouse-mab/14697, used in 188 publications), Anti-CD206 (Cell Signaling Technology, 24595, https://www.cellsignal.cn/products/primary-antibodies/cd206-mrc1-e6t5j-xp-rabbit-mab/24595, used in 156 publications), Anti-MMP13 (Proteintech, 18165-1-AP, https://www.ptgcn.com/products/MMP13-Antibody-18165-1-AP.htm, used in 465 publications), Anti-COL2A1 (Proteintech, 28459-1-AP, https://www.ptgcn.com/products/Collagen-Type-II-Antibody-28459-1-AP.htm, used in 254 publications). Santa Cruz Biotechnology states that Anti-IL-6 (Santa Cruz biotechnology, sc-57315, https://www.scbt.com/zh/p/il-6-antibody-10e5) detects IL-6 from mouse and rat sources by WB, IP, FCM, and ELISA.

# Eukaryotic cell lines

Policy information about cell lines and Sex and Gender in Research

Cell line source(s)

Raw 264.7 cells (#KGG2201-1), 293T cells (#KGG3101-1) were provided by keyGEN Biotech Inc., China. Synovial fluid-derived mesenchymal stem cells were a gift from Dr. Yujie Liang. Expi293F cells (#A14528) were acquired from Thermo Fisher Scientific, MA, USA. HcerEpic was procured from Xuanya Biotech (Shanghai, China, #XY1013) and cervical cancer cell line HeLa was purchased from Keygen (#KGG3232-1 ) .

Authentication

Cell lines were not authenticated.

Mycoplasma contamination

We confirm that all cell lines were negative for mycoplasma contamination.

Commonly misidentified lines
(See ICLAC register)

No commonly misidentified cell lines were used in this study.

# Palaeontology and Archaeology

Specimen provenance

Specimen deposition

Dating methods

☐ Tick this box to confirm that the raw and calibrated dates are available in the paper or in Supplementary Information.

Ethics oversight

Note that full information on the approval of the study protocol must also be provided in the manuscript.

# Animals and other research organisms

Policy information about studies involving animals; ARRIVE guidelines recommended for reporting animal research, and Sex and Gender in Research

Laboratory animals

The male C57BL/6 mice (18-22 g) and male SD rats weighing 190-210 g were purchased from Huachuang Sino and charles river. Mice

| | |
|---|---|
| Laboratory animals | and rats were maintained under specific-pathogen-free conditions in the animal facility of Southeast University. The animal room has a controlled temperature (18-24?), humidity (40-60%), and a 12 light/12 dark cycle. |
| Wild animals | No wild animals were used in this study. |
| Reporting on sex | Male mice or rats were used in our study and divided into different groups randomly. |
| Field-collected samples | No field-collected samples were used in this study. |
| Ethics oversight | All animal experiments were authorized by the Animal Ethics Committee of Southeast University under reference number 20220301031. |

Note that full information on the approval of the study protocol must also be provided in the manuscript.

# Clinical data

Policy information about clinical studies

All manuscripts should comply with the ICMJE guidelines for publication of clinical research and a completed CONSORT checklist must be included with all submissions.

| | |
|---|---|
| Clinical trial registration | |
| Study protocol | |
| Data collection | |
| Outcomes | |

# Dual use research of concern

Policy information about dual use research of concern

## Hazards

Could the accidental, deliberate or reckless misuse of agents or technologies generated in the work, or the application of information presented in the manuscript, pose a threat to:

| No | Yes | |
|---|---|---|
| ☐ | ☐ | Public health |
| ☐ | ☐ | National security |
| ☐ | ☐ | Crops and/or livestock |
| ☐ | ☐ | Ecosystems |
| ☐ | ☐ | Any other significant area |

## Experiments of concern

Does the work involve any of these experiments of concern:

| No | Yes | |
|---|---|---|
| ☐ | ☐ | Demonstrate how to render a vaccine ineffective |
| ☐ | ☐ | Confer resistance to therapeutically useful antibiotics or antiviral agents |
| ☐ | ☐ | Enhance the virulence of a pathogen or render a nonpathogen virulent |
| ☐ | ☐ | Increase transmissibility of a pathogen |
| ☐ | ☐ | Alter the host range of a pathogen |
| ☐ | ☐ | Enable evasion of diagnostic/detection modalities |
| ☐ | ☐ | Enable the weaponization of a biological agent or toxin |
| ☐ | ☐ | Any other potentially harmful combination of experiments and agents |

## Plants

| | |
|---|---|
| Seed stocks | N/A |
| Novel plant genotypes | N/A |
| Authentication | N/A |

## ChIP-seq

### Data deposition

☐ Confirm that both raw and final processed data have been deposited in a public database such as GEO.

☐ Confirm that you have deposited or provided access to graph files (e.g. BED files) for the called peaks.

| | |
|---|---|
| Data access links *May remain private before publication.* | |
| Files in database submission | |
| Genome browser session (e.g. UCSC) | |

### Methodology

| | |
|---|---|
| Replicates | |
| Sequencing depth | |
| Antibodies | |
| Peak calling parameters | |
| Data quality | |
| Software | |

## Flow Cytometry

### Plots

Confirm that:

☒ The axis labels state the marker and fluorochrome used (e.g. CD4-FITC).

☒ The axis scales are clearly visible. Include numbers along axes only for bottom left plot of group (a 'group' is an analysis of identical markers).

☒ All plots are contour plots with outliers or pseudocolor plots.

☒ A numerical value for number of cells or percentage (with statistics) is provided.

### Methodology

| | |
|---|---|
| Sample preparation | After plasmid transfection, cells were collected by centrifugation at 800 rpm to obtain 2 × 10^6 cells, which were then washed twice with pre-chilled PBS, followed by centrifugation at 800 rpm for 5 minutes each time. Untreated Expi293F cells served as the negative control. Subsequently, cell aggregates were removed by filtration through a 300-mesh nylon net, and the expression of EGFP fluorescent signal in the cells was analyzed by flow cytometry. |
| Instrument | Flow cytometric data were collected using FACS Calibur (BD Biosciences). |
| Software | FACS data was analyzed with FlowJo software (version 10.8). |
| Cell population abundance | 5000-10000 cells were counted per sample. |

Gating strategy: The predominant cell population on the FSC/SSC plot was gated.

☒ Tick this box to confirm that a figure exemplifying the gating strategy is provided in the Supplementary Information.

# Magnetic resonance imaging

## Experimental design

Design type

Design specifications

Behavioral performance measures

## Acquisition

Imaging type(s)

Field strength

Sequence & imaging parameters

Area of acquisition

Diffusion MRI ☐ Used ☐ Not used

## Preprocessing

Preprocessing software

Normalization

Normalization template

Noise and artifact removal

Volume censoring

## Statistical modeling & inference

Model type and settings

Effect(s) tested

Specify type of analysis: ☐ Whole brain ☐ ROI-based ☐ Both

Statistic type for inference

(See Eklund et al. 2016)

Correction

## Models & analysis

| n/a | Involved in the study |
|---|---|
| ☐ | ☐ Functional and/or effective connectivity |
| ☐ | ☐ Graph analysis |
| ☐ | ☐ Multivariate modeling or predictive analysis |

Functional and/or effective connectivity

Graph analysis

Multivariate modeling and predictive analysis
